# Probabilistic Inference for Nucleosome Positioning with MNase-Based or Sonicated Short-Read Data

**DOI:** 10.1371/journal.pone.0032095

**Published:** 2012-02-29

**Authors:** Xuekui Zhang, Gordon Robertson, Sangsoon Woo, Brad G. Hoffman, Raphael Gottardo

**Affiliations:** 1 Statistics Department, University of British Columbia, Vancouver, British Columbia, Canada; 2 Canada's Michael Smith Genome Sciences Centre, British Columbia Cancer Agency, Vancouver, British Columbia, Canada; 3 Vaccine and Infectious Disease and Public Health Sciences Divisions, Fred Hutchinson Cancer Research Center, Seattle, Washington, United States of America; 4 Child and Family Research Institute, British Columbia Children's Hospital and Sunny Hill Health Centre, Vancouver, British Columbia, Canada; 5 Department of Surgery, University of British Columbia, Vancouver, British Columbia, Canada; University of Jaén, Spain

## Abstract

We describe a model-based method, PING, for predicting nucleosome positions in MNase-Seq and MNase- or sonicated-ChIP-Seq data. PING compares favorably to NPS and TemplateFilter in scalability, accuracy and robustness to low read density. To demonstrate that PING predictions from widely available sonicated data can have sufficient spatial resolution to be to be useful for biological inference, we use Illumina H3K4me1 ChIP-seq data to detect changes in nucleosome positioning around transcription factor binding sites due to tamoxifen stimulation, to discriminate functional and non-functional transcription factor binding sites more effectively than with enrichment profiles, and to confirm that the pioneer transcription factor Foxa2 associates with the accessible major groove of nucleosomal DNA.

## Introduction

The structural unit for chromatin packaging is the nucleosome, which is composed of approximately 147 bps of DNA wrapped around a core histone octamer. Nucleosome-associated DNA is less accessible to regulatory proteins like transcription factors, and nucleosome positioning, as well as histone modifications and histone variants (e.g. H2A.Z, H3.3), are therefore influential in cellular processes that depend on chromatin accessibility [1]–[6]. Because nucleosome positions depend on cellular processes as well as intrinsic factors (e.g. DNA sequence), understanding how these positions influence cell states can require determining nucleosome locations within individual genomic regions [7].

Currently, genome-wide nucleosome-based data are typically generated by high-throughput short-read sequencing of DNA obtained by either MNase digestion (MNase-seq), or chromatin immunoprecipitation (ChIP-seq) of MNase-digested or sonicated DNA. MNase digests linker DNA with relatively high specificity [8], and this specificity is reflected in the narrow spatial distribution of aligned reads. However, sonication protocols are widely used; for example, in work to identify classes of functional genomic regions by integrated analysis of diverse sets of short-read sequence data [9]–[11].

Some methods proposed for inferring nucleosome positions from short-read data are heuristic and are based on simple pile-up profiles [12], [13]. While more elaborate approaches are available or have been described, such as NPS [14] and TemplateFilter (TpF) [15], or based on Hidden Markov Models (HMM) [16], these methods have been applied to data generated with protocols that use MNase-Seq, or MNase with ChIP-Seq (e.g. [17]), and their effectiveness with sonicated ChIP-seq data has not been demonstrated.

Recently we described PICS, a probabilistic peak-caller for identifying transcription factor binding sites in ChIP-Seq data [18]. PICS models bi-directional read densities, uses mixture models to resolve adjacent binding events, and imputes reads that are not mapped due to repetitive genome sequences. We anticipated that its model-based framework should be extensible to address both MNase-digested and sonicated nucleosome-based short-read data. We were interested in assessing how effectively the model could be adapted to the two data types, how robust the new algorithm would be to lower read densities, and the types of biological inferences that it would support from sonicated data. To address these issues, we developed PING, a method for probabilistic inference of nucleosome positioning from nucleosome-based sequence data. Like PICS, PING models bi-directional read densities, uses mixture models, and imputes missing reads. However, it uses a new prior specification for the spatial positioning of nucleosomes, has different model selection criteria, model parameters, and post-processing for estimated parameters. In addition, PING includes novel statistical methods to identify nucleosomes whose read densities are lower than those of neighboring nucleosomes.

In the work described here, we apply the new algorithm to three published short-read nucleosome-based data sets. We focus on regions around transcriptional start sites and *in vivo* transcription factor binding sites, which have well-defined nucleosome distributions [6], [19]. We demonstrate that PING performed well in identifying nucleosome positions in both MNase-Seq data in yeast and sonicated H3K4me1 ChIP-Seq data in mouse, and that it compares favorably to NPS and TpF in robustness to lower read densities. Then, using published data from a mouse cell line [20], we consider global changes in nucleosome positioning relative to *in vivo* binding sites for SPI1 (also known as PU.1) and CEBPB, and show that PING predictions from sonicated H3K4me1 ChIP-Seq data are consistent with published results from MNase-Seq data. Next, we apply PING to sonicated ChIP-Seq H3K4me1 data from mouse pancreas islet tissue [19]. We distinguish *in vivo* Foxa2 and Pdx1 binding sites that are between flanking H3K4me1-marked nucleosomes from sites that are within nucleosomal DNA. We show that genes associated with flanked TF-bound loci are more abundantly expressed than those associated with nucleosomal loci, consistent with flanked sites being active enhancer elements. Finally, we compare spatial distributions of binding sites on nucleosomal DNA for Pdx1 and for the pioneer transcription factor Foxa2.

## Results

In this section, we first describe PING's probabilistic model for inferring nucleosome positions from short-read sequencing data. Then, we compare the performance of PING, NPS and TpF, using three published datasets that have different experimental protocols and genome sizes: MNase-Seq data from budding yeast [21], sonicated ChIP-Seq data from a mouse cell line [20], and sonicated ChIP-Seq data from mouse pancreatic islets and liver tissue [19]. Finally, focusing on the data from mouse islet tissue, we demonstrate and assess several types of inferences from sonicated ChIP-Seq data.

### PING model

As in our previous work with transcription factor data [18], we first pre-process the read data by segmenting the genome into candidate regions, each of which has a minimum number of reads that aligned to forward and reverse strands. As in PICS, in each candidate region, conditional on the number of nucleosomes (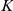) in the region, we model all the aligned read positions as independent and identically distributed (*iid*), as follows
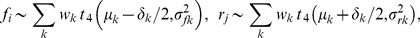
(1)where 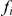 and 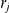 are the 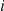-th forward and 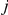-th reverse read positions in the region, with 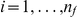 and 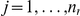, and 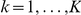 refers to the 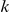-th nucleosome in the candidate region. The function 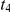 is the probability density function of a Student's t-distribution with four degrees of freedom. For the 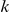-th nucleosome, 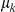 represents the position of its center, while 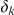 is the distance between the maxima of the forward and reverse read position densities, which corresponds to the average DNA fragment length in this bidirectional read cluster. Note that this length can differ from 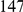 bp, as we discuss below for prior distributions. Because a DNA fragment should contribute a forward read or a reverse read with equal probability, we use a common mixture weight 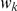 for both forward and reverse distributions. The parameters 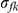 and 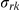 measure the corresponding variability in DNA fragment end positions. To accommodate possible biases related to sequencing and read mappability [18], [22] that result in asymmetric forward and reverse peaks, we do not assume or require that the forward and reverse variances of reads associated with a nucleosome are equal.

Since it models aligned reads as PICS does (1), PING inherits PICS' advantages, including robustness to outlier reads and imputation of missing reads (Methods). PING's main novelty is in the modelling of nucleosome positions and their downstream inference, as explained below.

**Figure 1:**
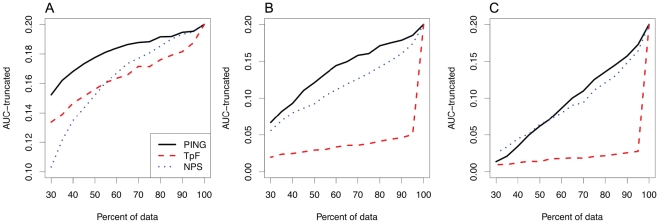
Truncated AUC statistics for PING, TpF and NPS. Panels show the area under ROC curves (AUC), truncated at a specificity of 0.8, as a function of number of reads in random subsets for PING, TpF and NPS. A larger AUC value corresponds to a more accurate method; the maximum possible AUC value for the truncated curves is 0.2. Datasets are (A) MNase-Seq data from budding yeast [21], (B) sonicated H3K4me1 ChIP-Seq data from a mouse cell line [20], and (C) sonicated H3K4me1 ChIP-Seq data from mouse adult islet tissue [19].

In PING, the nucleosome positions (the 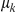
's) are assumed, a priori, to be drawn from a one dimensional Gaussian Markov random field (GMRF) distribution [23]. GMRF distributions are well suited to modelling the linear arrays that are typical of nucleosomes. The prior distribution of 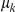
's is defined conditionally on neighboring nucleosomes as
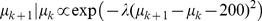
(2)where 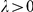 is a fixed parameter. This prior states that consecutive nucleosome centers should be separated by approximately 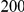 bp. A larger 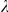 value will constrain distances to be closer to 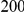 bp, while a smaller value will allow a wider range of values. After characterizing the effect of 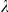 on the prior, we chose a relatively weak prior by setting 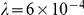, which corresponds to a distance between adjacent nucleosomes of between 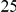 and 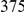 bp. The lower bound permits detecting nucleosome positions that are closely spaced due to positioning varying between sub-populations of cells [8], while the upper bound accommodates short nucleosome-free regions. Note that segmentation into candidate regions excludes genomic regions with low read densities that are longer than 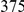 bp from candidate regions. Figure 1 in ‘figure S1’ shows an example of random samples from this prior.

**Figure 2:**
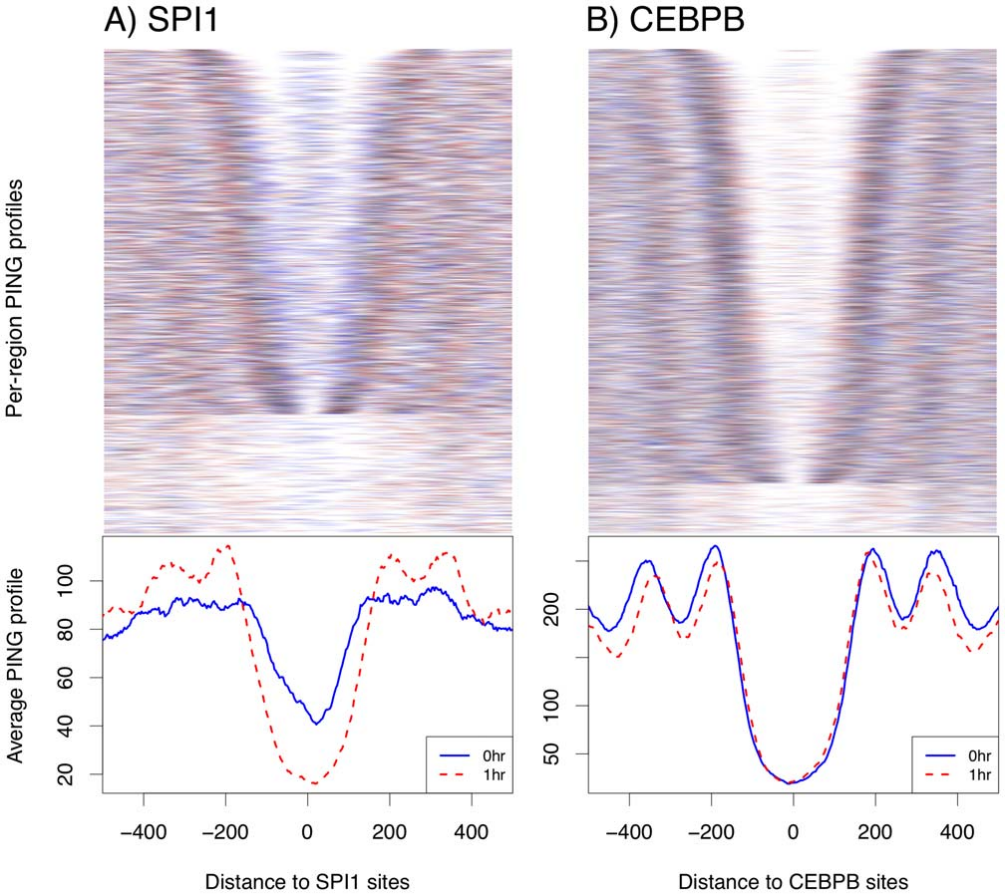
Model-based nucleosome occupancy profiles for sonicated H3K4me1 ChIP-Seq data. Panels show nucleosome positioning within 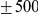 bp from the top-ranked 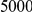 *in vivo* transcription factor binding sites that PICS detected for (A) SPI1 and (B) CEBPB from sonicated H3K4me1 ChIP-Seq data for 0 hour (blue) and 1 hour (red) after tamoxifen stimulation [20]. The heatmaps show nucleosome prediction profiles for each region as pairs of blue/red horizontal lines, with darker colors indicating higher scoring, i.e. better positioned, nucleosomes. The lower part of each heatmap shows genomic regions that lack detectable nucleosome positioning. Curves below each heatmap show average occupancy profiles across all TF regions.

The remaining parameters 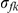, 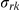 and 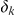 summarize our prior knowledge about the DNA fragment size distribution. For computational convenience we use a Normal-Gamma conjugate prior defined by
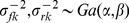
(3)

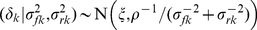
(4)where 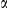, 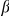, 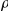, and 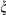 are fixed hyper-parameters. Such conjuate priors are commonly used in hierarchical Baeysian modeling for genomic data because they lead to closed form iterative algorithms for posterior exploration [24], [25]. In our context, 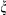 represents our best prior guess about the mean fragment length distribution across nucleosomes, while 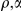 and 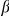 control the spread around this guess. For data generated by an MNase protocol, we set 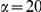, 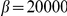, 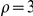, and 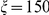, which result in 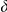 values between 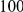 and 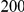 bp (figure 2 in ‘figure S1’). For data generated by a sonication protocol, where we expect DNA fragment lengths to be more variable, we used 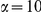 and 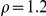, which result in 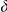 values between 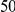 and 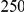 bp. The parameters were chosen empirically from exploratory analyses on several ChIP-seq and MNase-seq samples, and from our knowledge of the library construction for the experiments. Parameter values can be adjusted by a user, given, for example, different fragment lengths from library construction (see ‘text s1’).

### Methods comparison

Because the cost of sequencing experiments can constrain work involving large genomes and experimental designs, we evaluated the performance of PING, NPS [14] and TpF [15] over a range of sequencing depths, using the three data sets noted above. Two considerations led us to generate test datasets by subsampling rather than simulation. First, it was not clear how to simulate data in which both the position and number of reads for a nucleosome may depend on neighboring nucleosomes. Second, the MNase-Seq yeast data were deeply sequenced, given the compact genome. For these data, many nucleosomes had strong, well-defined read signals, and many appeared to be both well positioned and accurately predicted by all three methods (see ‘examples S1’). This suggested that at least this dataset would give good ‘reference’ nucleosome positions for the subsampling comparisons.

**Figure 3:**
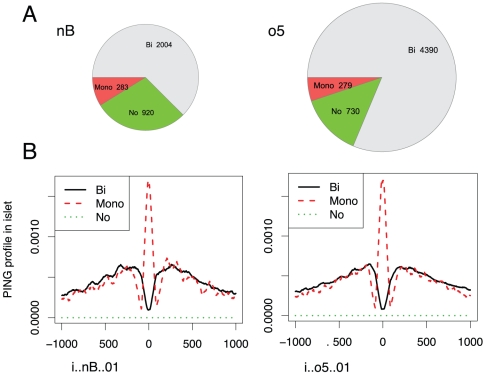
Modality and nucleosome occupancy for Foxa2 and Pdx1 binding sites in mouse adult islet tissue. Panels show the modality and nucleosome profiles for *in vivo* binding sites of the transcription factors Foxa2 (left) and Pdx1 (right) [19]. (A) The number of binding sites in bimodal(bi), monomodal(mono) and NoNuc(No) groups. A NoNuc transcription factor binding site had no H3K4me1-marked nucleosome within 1 kb of its peak summit, a monomodal site had at least one H3K4me1 nucleosome within 50 bp of its summit, and all other sites were bimodal. (B) Average model-based nucleosome positioning profiles for the three classes of binding sites.

**Figure 4:**
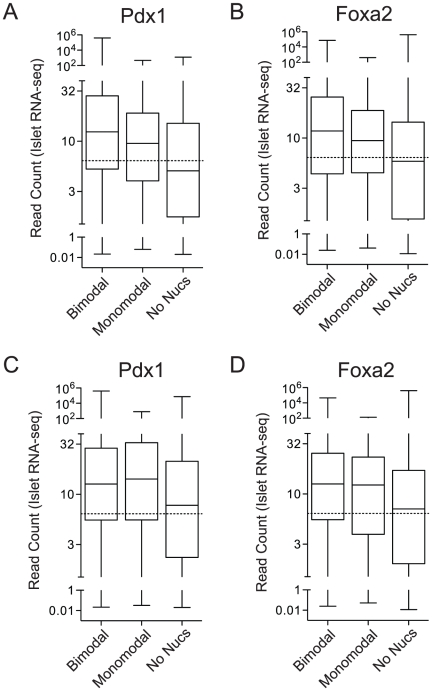
Expression levels for genes associated with different types of nucleosome predictions. RNA-seq data for mouse adult islets are from [29]. Nucleosomes were predicted from H3K4me1 data for (A,B) mouse adult islets and (C,D) mouse adult liver [19]. Dashed horizontal lines show medians. In islets, genes categorized as bimodal and NoNuc respectively have significantly higher and lower expression levels than those in the monomodal group. Nucleosome prediction groups are outlined in Fig. 3's caption and in Methods.

**Figure 5:**
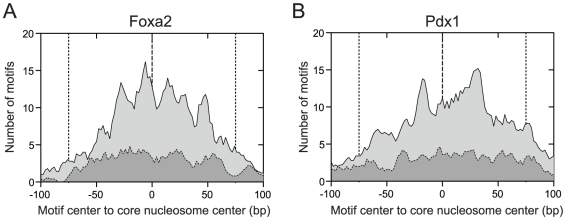
Profiles of predicted Foxa2 and Pdx1 binding sites on nucleosomal DNA. Panels show profile of predicted transcription factor binding sites of (A) Foxa2 and (B) Pdx1 closest to the centre of a predicted PING nucleosome position for monomodal binding sites in mouse islet tissue [19]. Dashed curves above dark gray regions show corresponding profiles in mouse liver. Profiles are truncated at 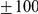 bp, and vertical dashed lines show 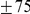 bp from the estimated centres of the nucleosome-associated DNA. Foxa2 binding sites are enriched near the nucleosome centers and show a periodicity of 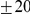 bp, while Pdx1 binding sites are enriched at two locations that are 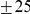 bp from the nucleosome center.

For Kaplan's MNase-Seq yeast data we used the most deeply sequenced sample, NOCL4. For the two mammalian datasets, we considered a subset of regions that were associated with transcription factor binding sites, and so should have relatively well-positioned nucleosomes [17], [19], [26]. For the mouse PUER cell line data [20] we selected the 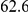 thousand MNase-Seq reads that were within 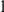 kb of centers of the top-ranked 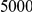 CEBPB-enriched regions detected by PICS for 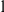 hour after treatment with tamoxifen. For mouse islet data we selected the 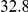 thousand H3K4me1 sonicated ChIP-Seq reads that were within 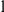 kb of centers of the top-ranked 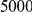 Pdx1 peak summits [19]. As reference nucleosomes we selected 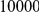 top-ranked nucleosomes of each method in the yeast MNase-Seq data and 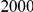 top-ranked nucleosomes of each method in the mouse sonicated ChIP-Seq data (figures 3, 4, and 5 in ‘figure S1’).

For each of the three data sets, we generated 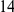 random subsets of reads that contained from 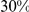 to 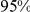 (with step size 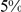) of the original number of reads. For each data subset we calculated area-under-the-curve (AUC) statistics for the three methods (Methods). A larger AUC value for a subset of reads indicates that reference nucleosome positions were detected more accurately and frequently.

Figure 1 shows AUC profiles as a function of the number of reads in random subsets for three methods. AUCs for PING were consistently larger than for three methods, suggesting that PING can predict nucleosome positions more accurately and may require less deeply sequenced data than the other two methods. TpF showed comparable performance only in Kaplan's MNase-Seq data, for which its templates should be appropriate (see ‘examples S1’). NPS predicted only nucleosomes that had relatively high read counts; while our comparison method is favorable to NPS, this method's performance was lower with the larger sets of reference nucleosomes, for which it returned maximum sensitivities less than one.

Tests with alternative settings showed that results were robust to the number of nucleosomes in reference sets. For example, we tried reference sets with 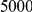 or 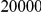 nucleosomes in Kaplan's data, and reference sets with 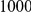 or 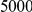 nucleosomes in Heinz's and Hoffman's data. In these assessments, PING generally returned larger AUCs than the other methods (data not shown).

Prior to sequencing, given the biology that an experimental design will address, it is desirable to be able to estimate how deeply a sample should be sequenced; given sequencing data, it is desirable to be able to estimate whether sufficient sequence data has been generated. The AUC approach shown here may be appropriate way to address the second issue, as, for an experiment in which the slope of the curve is low as it approaches 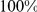 of the reads available, additional reads are unlikely to improve the results.

### Inferring nucleosome positioning with sonicated ChIP-Seq data

As noted above, much histone modification data is available from protocols in which the DNA has been fragmented by sonication. In this section, using data for a mouse cell line, we assess nucleosome-level results generated by PING from sonicated data, and compare these with published occupancy profiles from MNase-Seq data. We considered four biological states: before vs. 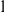 hour after tamoxifen stimulation, and regions around SPI1 vs. CEBPB binding sites.

Using MNase-Seq data from PUER cells, in which SPI1 becomes localized to the nucleus and can bind DNA only after tamoxifen treatment, Heinz et al. [20] showed that, globally, positions of nucleosomes flanking SPI1 binding sites are more distant from SPI1 sites after SPI1 binding. To determine whether we could use PING to generate similar results using sonicated ChIP-Seq data, we predicted nucleosome positions genome-wide from sonicated ChIP-Seq H3K4me1 samples for both 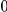 hour and 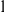 hour after tamoxifen stimulation, and used PICS to predict binding sites for SPI1 and for CEBPB. Figure 2 shows model-based nucleosome profiles in 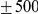-bp regions around the top-ranked 5000 binding sites for both factors (compare to figure 9 in ‘figure S1’). The heatmaps show individual regions as pairs of blue/red horizontal lines (denoting 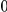 and 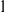 hr respectively), with darker colors indicating higher scoring (i.e. better positioned) nucleosomes, while the profiles show the average nucleosome occupancy across all TF binding regions. For both transcription factors and time points, the heatmaps show that the distance between 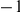 and 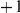 nucleosomes varies between regions, suggesting caution in interpreting average profiles alone. Despite this, from the heatmaps and profiles it is evident that the closest three nucleosomes have shifted away from SPI1 binding sites by 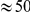-bp at the 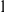 hr time point, consistent with MNase-Seq data (Figure 4D in [20]). We note that while the published MNase-Seq profile more clearly indicates a global 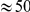-bp shift, the relatively low MNase-seq read densities did not support model-based nucleosome predictions on individual genomic regions. Also, because SPI1 is not localized to the nucleus at 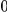 hr [13], its profiles at this time point are poorly defined; in contrast, upon SPI1 binding, at 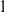 hr, the nucleosome profiles are better defined, suggesting that binding stabilizes flanking nucleosome positions. Both heatmaps and profiles suggest that nucleosome positioning is well defined for CEBPB at both time points. While a small positional shift was evident for this factor, CEBPB is localized in the nucleus and so is expected to be associated with DNA at both time points, and this result is of uncertain biological significance.

Together, these data from a mouse cell line indicate that PING can be effective in inferring changes in nucleosome positions with sonicated ChIP-Seq data, but that the degree of positioning, and so the inferences possible, can depend on the transcription factor and on the biological state.

### Identifying transcription factor-nucleosome interactions in mouse islet data

Transcription factor binding sites typically occur within nucleosome-free regions flanked within 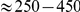 bp by H3K4me1-marked nucleosomes (‘bimodal’ sites) ([17], [19], [26]). Hoffman (2010) used enrichment profiles [27] to show that both Pdx1 and Foxa2 can also bind motifs within regions enriched for H3K4me1 (‘monomodal’ sites). Such a pattern of association is characteristic of ‘pioneer’ transcription factors (TFs) like Foxa2 [28]. Comparing the functional properties of *in vivo* Pdx1 and Foxa2 binding sites that were in bimodal vs. monomodal regions indicated that only bimodal Pdx1- and Foxa2-bound loci were functional in regulating gene expression.

To determine whether PING-based nucleosome predictions could be used to distinguish transcription factor binding sites flanked by paired H3K4me1-marked nucleosomes from sites within nucleosomal DNA, we applied PING to the sonicated H3K4me1 ChIP-Seq data from mouse adult islets and liver (Methods). We used the resulting predicted nucleosomes in islets to classify *in vivo* binding sites of Pdx1 and Foxa2 in islets, using nucleosomes that we predicted in H3K4me1 data from mouse liver as a negative control. From the spatial relationship between a TF binding site (taken as the summit of an enrichment profile peak) and nearby predicted nucleosomes in islets, we classified binding sites into three subgroups: those flanked by paired H3K4me1-marked nucleosomes (‘bimodal’), those within H3K4me1-marked nucleosomal-DNA (‘monomodal’), and those with no H3K4me1-marked nucleosomes within 1kb (‘NoNuc’). Figure 3a shows the number of regions in each group, and shows that, consistent with the data of Hoffman et al, the majority of transcription factor binding sites (62–81%) are bimodal; however, between 5 and 9% are within nucleosomal DNA.

Figure 3b shows the average read density profiles for sites in the three subgroups, and the average model-based nucleosome positioning profiles for sites flanked by paired nucleosomes versus those bound to nucleosomal DNA. In the center of the regions, at the Pdx1 and Foxa2 peak summit locations, profiles show a deep valley for sites identified as flanked by paired H3K4me1-marked nucleosomes, a sharp peak in the read density profile for sites identified as bound within nucleosomal DNA, and a flat unenriched profile for the “NoNuc” group. As a negative control, we show the same profiles generated from mouse adult liver, in which Pdx1 is not expressed, and in which Foxa2 binds 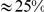 of the sites identified in islets [19] (Figure 8 in ‘figure S1’). In liver, the sites identified as flanked by paired nucleosomes show a slightly lower nucleosome density at Pdx1 peak summit locations, suggesting that some of these loci are bound by other factors in this tissue. In contrast, the sites identified as bound within nucleosomal-DNA for Pdx1 have no distinct profile. For Foxa2 some reduction in nucleosome density is noted at the peak summit location for sites identified as bound within nucleosomal-DNA in islets, probably because some of these sites are bound in liver. This is consistent with previous results indicating that Foxa2 loci that are bound in both islets and liver, and are monomodal in one tissue, are often bimodal in the other [19]. Note that compared to Heinz's data, these data were generated from tissue rather than a cell line; hence, we may expect more biological heterogeneity and variability, and so potentially more variability in nucleosome positions.

Following Hoffman (2010), to assess our classification results using independent data we compared the expression levels for genes associated with subgroups of binding regions, using published islet RNA-seq data [29] (Figure 4). We assessed expression levels differences between groups using a Kruskal-Wallis test and a null hypothesis that there is no difference among gene expression levels of three groups vs. the alternative hypothesis that at least two groups are different. P-values were less than 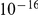 for all combinations of two transcription factors and two tissues. We then conducted a post-hoc multiple pairwise comparison [30] for each combination of group pairs. Genes associated with loci that lacked H3K4me1-marked nucleosomes were significantly less expressed than regions in other groups (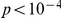). In contrast, genes associated with loci within nucleosomal DNA were significantly less expressed than genes associated with loci flanked by paired nucleosomes (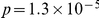 for Pdx1 and 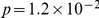 for Foxa2). This is consistent with sites flanked by paired nucleosomes being more functionally active. As expected, we saw no difference between these site types using H3K4me1-based nucleosome calls in liver, using the same islet RNA-seq data (p = 0.99 for Pdx1 and p = 0.21 for Foxa2). These results show that using nucleosome positions predicted by PING to define the ‘modality’ of transcription binding sites generates more effective bi/monomodal classification results than those originally generated from enrichment profiles.

Unlike Foxa2, Pdx1 is not known to be a pioneer factor, i.e. a factor that can bind motifs within nucleosomal DNA [19]. Given this, we compared spatial distributions of Foxa2- and Pdx1-bound sites at predicted nucleosome locations to assess where these transcription factors were predicted to bind within the nucleosomal DNA. By profiling the density of the *de novo* Foxa2 and Pdx1 binding sites that were closest to their respective peak summit locations, we found that Foxa2-bound sites were enriched near the nucleosome centers and showed a periodicity of 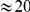 bp (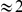 helix turns) (Figure 5). This profile is consistent with Foxa2 binding at locations where the major groove faces away from the histone octamer, as expected for its helix-turn-helix domain [27], [31]–[33]. In contrast, Pdx1-bound sites were enriched at two locations that were 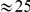 bp from the nucleosome center. While Pdx1's homeo domain also associates with the major groove [34], the site profile for this factor was only partially consistent with the locations in which the major groove is accessible. To confirm that these patterns of enrichment were a result of constraints placed on Pdx1 and Foxa2 binding, we found no comparable spatial enrichment of Pdx1 and Foxa2 motifs around monomodal sites identified using nucleosome positions predicted from liver H3K4me1 data (Methods). These results indicate that both Pdx1 and Foxa2 can bind within nucleosomal DNA, but have different preferences for binding locations within the nucleosome. As well, the results confirm that, for appropriate biological states, nucleosome positioning can be defined with high spatial resolution from sonicated data.

## Discussion

In the work reported here, we describe PING, a model-based method for predicting nucleosome positioning that can flexibly be applied to either MNase-based and sonicated ChIP-Seq data. Using an sampling-based ROC/AUC analysis, and three data sets with different characteristics, i.e. MNase-Seq data from budding yeast, and sonicated ChIP-Seq H3K4me1 data from a mouse cell line and from mouse islet tissue, our method showed better overall predictive accuracy and scalability than NPS and TemplateFilter. While additional methods have been described, the two methods that we used in comparisons have been shown to perform well, and offer reasonable performance baselines. These comparisons also showed that PING can readily be applied to data from mammalian genomes, and is relatively robust to low read densities.

Given a method that could be applied to both MNase-based or sonicated data, we addressed the question of the spatial resolution available from sonicated data. Using published sonicated H3K4me1 ChIP-Seq read data in a mouse cell line, PING-based results for nucleosome displacement away from transcription binding sites after tamoxifen stimulation were consistent with results reported for MNase-Seq data. However, the contrast between the occupancy profiles for SPI1 and CEBPB indicated that nucleosome positioning predictions can depend on the biological state.

Using PING-based nucleosome predictions from sonicated H3K4me1 ChIP-Seq data from mouse islets, we refined the classification of *in vivo* Foxa2 and Pdx1 binding sites into three groups, and showed that the between-group gene expression differences were more statistically significant for the updated groups. Characterizing the binding profile of the pioneer transcription factor Foxa2 on nucleosomal DNA in islet tissue, we showed that, for appropriate biological states, sonicated data can support positioning predictions that have high spatial resolution. These results, and the flexibility and scalability of the PING method, suggest that it may be useful in generating mechanistic insight within sets of individual genomic regions using short-read data; for example, in regions in which specific combinations of epigenetic marks are associated with particular functional properties.

## Materials and Methods

### Data sets

The ‘Kaplan’ MNase data are from *S. cerevisceae* (GEO data set GSM351492 [21]). They were generated using MNase-Seq, i.e. digesting linker DNA with MNaseI, size selecting mononucleosome DNA fragments, and single-end sequencing the ends of these fragments. There are six biological replicates. Four have no formaldehyde cross-linking and have between 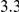 and 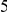 million aligned reads; two were cross-linked and have between 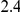 and 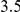 million aligned reads. These samples were deeply sequenced, given the compact 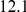 Mb genome. Many nucleosomes had strong, well-defined aligned read signals, and many appeared to be both well positioned and accurately predicted by all three methods (see ‘examples S1’).

The sonicated ChIP-Seq ‘Heinz’ data were generated from the mouse PUER cell line [20]. We used two H3K4me1 sonicated samples from GEO data set GSE21512, which corresponded two biological states: 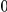 hour, i.e. before stimulation (GSM538012) and 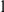 hour after tamoxifen stimulation (GSM538013). Single-ended 25-bp reads were generated after sonicating chromatin to 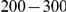 bp, then immunoprecipitating with an antibody against H3K4me1 (Abcam ab8895). These two samples contained 8.1 and 7.2 million aligned reads respectively, and, given the 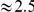 Gb mouse genome, they were much less deeply sequenced than Kaplan's data.

The sonicated H3K4me1 ChIP-Seq ‘Hoffman’ data were generated from mouse adult pancreas islet and liver using Abcam ab8895, as described in [19]. The data contains 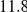 and 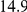 million aligned reads in islet and liver respectively.

For Heinz data, to obtain the *in vivo* binding sites of transcription factors of SPI1 and CEBPB, we used PICS [18] to analyze the ChIP-Seq data GSM538000 (SPI1, 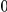 hr), GSM538001 (SPI1, 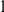 hr), GSM538006 (CEBPB, 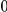 hr), and GSM538007 (CEBPB, 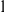 hr). The *in vivo* binding sites for the transcription factors of Pdx1 and Foxa2 were obtained from [19].

### Filtering duplicated reads

A relatively high number of duplicate reads (i.e. single-end reads with identical 5′ alignment coordinates) may be the result of biases in library construction and PCR amplification. Since the spatial distributions of locations of fragment starts should be more concentrated near ends of wrapped DNA for MNase than for sonicated data, we expect that we could see more repeated reads that are *not* process artifacts in MNase data. To control potential artifacts, while accommodating differences between MNase vs. sonication protocols, we removed reads beyond an upper bound that is set as a quantile for the number of duplicates found while processing a data set. In practice, we chose the 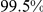 quantile for MNase data, and the 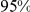 quantile for sonicated data. These threshold quantiles can be set by a user. And, since PING models read densities, highly ranked nucleosome predictions should be rather insensitive to the value set for the upper bound on duplicates.

### Segmentation of candidate regions

To process large data sets, particularly when multiple CPU cores are available, it is preferable to split the aligned read data into smaller disjoint subsets and process each subset separately. We first segment the genomic aligned read data into ‘candidate’ regions, each of which has a minimum number of reads that were mapped to forward and reverse strands. The segmentation step is similar to that used for PICS [18]. To suit nucleosome-based data, we adjusted the parameters and added an additional recursive splitting step to avoid candidate regions being too long.

We detect such regions using a 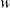-bp sliding window with an 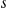-bp step size, counting the number of forward and reverse strand reads in the left and right half-windows respectively (reads within 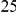 bps from the center of the window are not counted), and we retain windows that contain at least 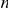 forward reads and 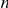 reverse reads. Here we used 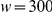 bp, 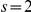 bp and 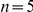 and merged overlapping windows from left to right to obtain a disjoint set of candidate regions (parameters are discussed in ‘text S1’).

Depending on the density of nucleosomes expected across the genome for a given experiment (e.g. MNase-Seq), segmentation could result in genomic regions that are long enough that applying a mixture model to infer nucleosomes requires extended computing times. To avoid this, we recursively divide candidate regions that are longer than 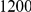 bps at points of low read density, until no regions are longer than this.

### Parameter estimation and model selection

Given the conjugate prior chosen, an Expectation-Maximization (EM) algorithm can be derived to find the maximum a posteriori (MAP) estimates for the unknown parameter vector 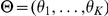, where 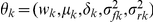. Our algorithm is similar to that used in PICS [18], and it is described in detail in the ‘text S1’. The main difference comes in the M-step, for which we developed a novel procedure to incorporate the spatial prior for the 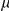
's.

### Model fitting

After segmenting the whole genome into candidate regions using a sliding window, we fit a PING model in each candidate region. In practice, 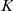, the number of mixture components in each region, is unknown and needs to be estimated. In our previous work with ChIP-Seq data for transcription factors (TFs), we used the Bayesian Information Criteria (BIC) to estimate the number of components, by trying 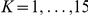 and selecting the value of 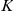 with the largest BIC. For nucleosome-based sequence data, candidate regions are often longer than is typical for TF data, and we expect to routinely encounter much larger values of 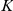 than for TF data. To reduce computing time, we try only integer values of 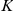 in the interval 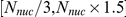 where 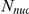 is the expected number of nucleosomes in a region, given the region's length, and is calculated as 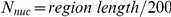. Note that this range will vary from region to region and is dynamically set during a run.

### Choosing the number of nucleosomes in each region

After having fit a model for each value of 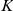 in the above range, we need to select a single best value in order to make inferences about the nucleosome positions. While BIC is well suited for selecting the number of components in mixture models, it does not effectively use the information contained in our spatial prior (Eq. 2). Instead, we use a log likelihood penalized by our prior for 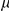. We select the value of 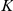 with the largest penalized log likelihood as follows,
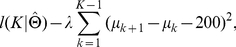
(5)where 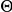 is the final estimate for the parameters 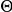, and *l* is the log-likelihood as defined in the ‘text S1’. Even though our model selection procedure gives satisfactory results for most regions, we noted a few cases in which the results were not optimal because of noise in read distributions. As with our PICS model, we have derived approaches to check for noisy estimates and wrongly estimated values of 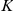, and to correct for these if needed. See ‘text S1’ for details.

### Scores of predicted nucleosomes, false discovery rates, and differential enrichment of nucleosomes in two high-throughput sequencing samples

In order to identify and rank a statistically meaningful subset of nucleosomes, we define an enrichment score for each nucleosome. For a given nucleosome, we define 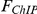 (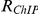), the number of observed forward (reverse) ChIP read positions that fall within the 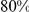 contours of the forward (reverse) read position densities, i.e. within 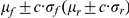 where 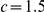 (approximately the 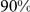 quantile of the 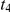 distribution). We then define the enrichment score as 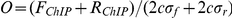, which is an estimate of the observed density of DNA enriched fragments contributing to this nucleosome, after removing outliers. When a control sample is available, we also define 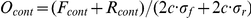, by computing the number of observed forward/reverse reads in the control sample that fall within the 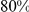 contour of the forward/reverse read position densities estimated from the ChIP sample. Using this information, we define an enrichment score for the treatment relative to the control as 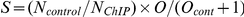, where the addition of the constant one prevents a division by zero, and 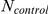 (resp. 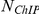) denotes the total read count in control sample (resp. IP sample). The scaling of the enrichment score by 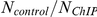 accounts for the control and ChIP samples having different numbers of reads (sequence depth). Note that the score introduced here is slightly different from the one used in PICS [18]. We made improvements by normalizing the scores by their peak widths (sigmas) which produces more stable nucleosome-based scores. When control sample is available, false discovery rates (FDR) can be calculated from the scores of nucleosomes using the approach proposed in PICS [18].

Note that the estimated FDR depends on the definition of a false call, and so on the choice of the negative control data. For the work described here, we had available input DNA for the pancreas islet and liver data sets [19]. Input DNA is widely used in protein-DNA association work (i.e. transcription factor binding); however, PING returned 475108 nucleosome calls from H3K4me1 data, but only 1281 from the control dataset. So, while input DNA should be useful for filtering out ChIP-seq artifacts, it appears to be less useful for estimating an FDR in the context of nucleosome prediction. H3/H4 datasets have been used to normalize histone modification profiles against nucleosome density profiles (e.g. [35]). Sequencing datasets from H3/H4 should have more appropriate aligned read profiles than input DNA, and so may be useful for estimating FDRs for nucleosome predictions; unfortunately, these were not available for the data used here.

### Calculating AUC values

We calculated AUC values in four steps. First, we predicted nucleosomes using PING, NPS and TpF; i.e. we generated three method-specific sets of reference predictions. The number of predicted nucleosomes in each random subset is given in ‘table S1’. Second, we generated receiver operating characteristic (ROC) curves for each method using the predicted and reference nucleosomes. Third, we truncated each ROC curve at a specificity of 0.8, since sensitivity is of little value without a reasonable specificity. Finally, we calculated AUC statistics as the area under these truncated ROC curves.

To generate an ROC curve, we needed to define a threshold distance, so that a reference nucleosome is called ‘detected’ if the distance between the centre of the reference nucleosome and a nucleosome predicted from a subset of reads is less than the threshold. The threshold distances were chosen as 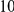 bp in Kaplan's MNase data, 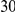 bp in Heinz's and Hoffman's sonication data. These values resulted in the areas under most full ROC curves being larger than 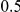, where 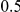 is expected value for binary random guesses. When we tested a distance that resulted in all methods detecting reference nucleosomes less accurately than a binary random guess, we increased the distance. To assess how robust our results were with respect to the threshold distance, we tried alternative settings and noted similar results. For example, we tried threshold distances of 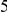 bp in Kaplan's MNase data, 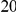 bp and 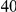 bp in two sonication data as well as AUC statistics calculated from the full ROC curves instead of the truncated ones. In all of these assessments, PING generally performed better than the two other methods.

### Classification of transcription factor binding regions

We classify binding regions according to the distances between a TF binding site to the nearest called nucleosome. After removing weak nucleosome calls (see the following subsection for details), we classify regions as follows. A binding region without any H3K4me1-marked nucleosome detected within 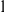 kb of its peak summit is called a “NoNuc” region. A binding region with at least one H3K4me1 nucleosome detected within 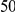 bp of its peak summit is called “monomodal”. Other binding regions are called “bimodal”.

### Removing nucleosomes that have low read densities

Because nucleosomes with relatively low read densities are more likely to be falsely called as present, it is helpful to detect and remove them from PING predictions. For this, we compare each predicted nucleosome to other nucleosomes in its neighborhood, as follows.

For each predicted nucleosome, referred to as the ‘reference nucleosome’, we select other predicted nucleosomes within 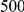 bp. We ignore any nucleosomes that are separated from the ‘reference nucleosome’ by a nucleosome-free region longer than 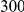 bp, which is PING's upper threshold for filtering estimated 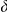
's. We refer to these selected nucleosomes as ‘neighborhood’ nucleosomes. We compare the reference nucleosome to each of its neighborhood nucleosomes, and consider the reference nucleosome as ‘falsely-called’ if its read count is significantly lower than that of any neighborhood nucleosome. In these comparisons, a read count ratio for two nucleosomes is significantly different if it is higher than a threshold, which we calculate adaptively using a negative binomial model that takes into account the widths (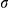) of forward/reverse read density distributions of the nucleosomes, as follows.

In a neighborhood, given the reads count (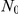) of a reference nucleosome, the reads count (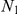) of another nucleosome in its neighbourhood follows a negative binomial distribution
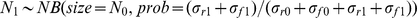
where 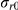 and 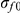 describe the width of forward/reverse peak of reference nucleosome, and 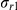 and 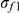 describe the width of forward/reverse peak of the nucleosome to be compared with. An example threshold curve of 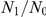 is given in figure 6 in ‘figure S1’.

### Multiple transcription factor binding regions associated with the same gene

Multiple transcription factor binding regions can be associated with the same gene. TFBS sites that are flanked by H3K4me1-enriched regions are functional, while sites within H3K4me1-enriched regions, or in regions without H3K4me1, are non-functional in regulating gene expression [19]. Given this, when we identify a monomodal region for a gene, we ignore NoNuc regions for the same gene, and when we identify bimodal regions, we ignore both monomodal and NoNuc regions for that gene.

### Distribution of Pdx1 and Foxa2 motifs around monomodal sites identified using liver nucleosome positions

While we were interested in the results in islets, we also generated the same results from liver data as a negative control, to show that the results obtained in islet data were unlikely to have occurred by chance. To generate the liver results, we needed to identify nucleosome predictions that overlapped with a TF peak summit in liver. For this, we use predicted nucleosomes from liver H3K4me1 data to classify islet transcription factor binding sites and obtained liver “monomodal” sites and corresponding liver core nucleosomes.

In each “monomodal” region, we determined the TF site closest to the peak summit of the transcription factor binding region, and considered the center distance of this motif to the center of the central nucleosome. We considered all “monomodal” regions, as well as a subset of them chosen from the regions whose central nucleosomes had corresponding PING score in the top 50000 among all predicted nucleosomes whole genome, again using the elbow point of score distributions of all whole-genome predicted nucleosomes (figure 7 in ‘figure S1’).

## Supporting Information

Figure S1 **Contains all supplementary figures referred in the main manuscript.**
(PDF)

Table S1 **Contains the supplementary table referred to in the main manuscript.**
(PDF)

Text S1 **Describes details of methods, and discusses PING's parameters.**
(PDF)

Examples S1 **A multi-page figure showing details of PING, NPS and TemplateFilter nucleosome calls in several genomic regions.**
(PDF)
