# Supplementary material for: Probabilistic Inference for Nucleosome Positioning with MNase-Based or Sonicated Short-Read Data
Source: PLoS One. 2012 Feb 29;7(2):e32095. doi: 10.1371/journal.pone.0032095 (PMC3290535; doi:10.1371/journal.pone.0032095)
Supplement: Figure S1 — Contains all supplementary figures referred in the main manuscript. (PDF) [file pone.0032095.s001.pdf]

Supplementary figures for: Probabilistic Inference for Nucleosome Positioning with MNase or Sonicated Short-read Data

1. Choosing values for hyperparameters

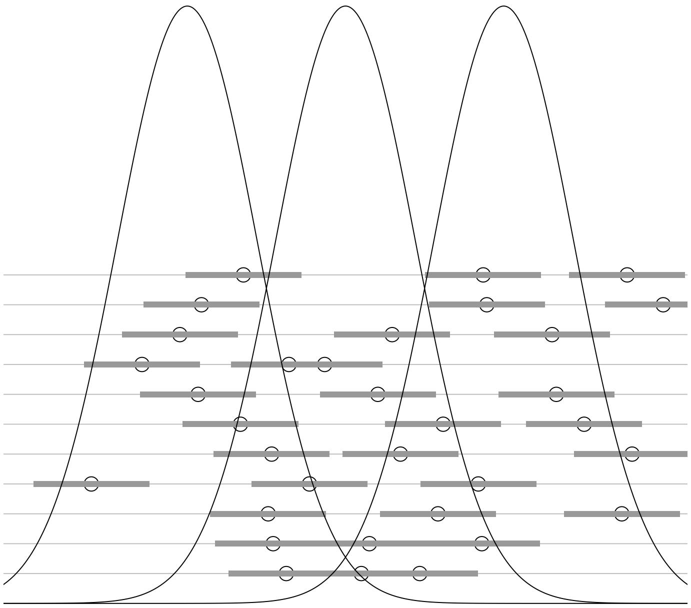

Figure 1. Density of the Gaussian Markov random field (GMRF) used in PING, with pale horizontal lines showing 10 simulations or realizations of 3 adjacent nucleosomes from this prior. The darker fragments on each line shows the three simulated nucleosomes of one realization; circles show their centers.

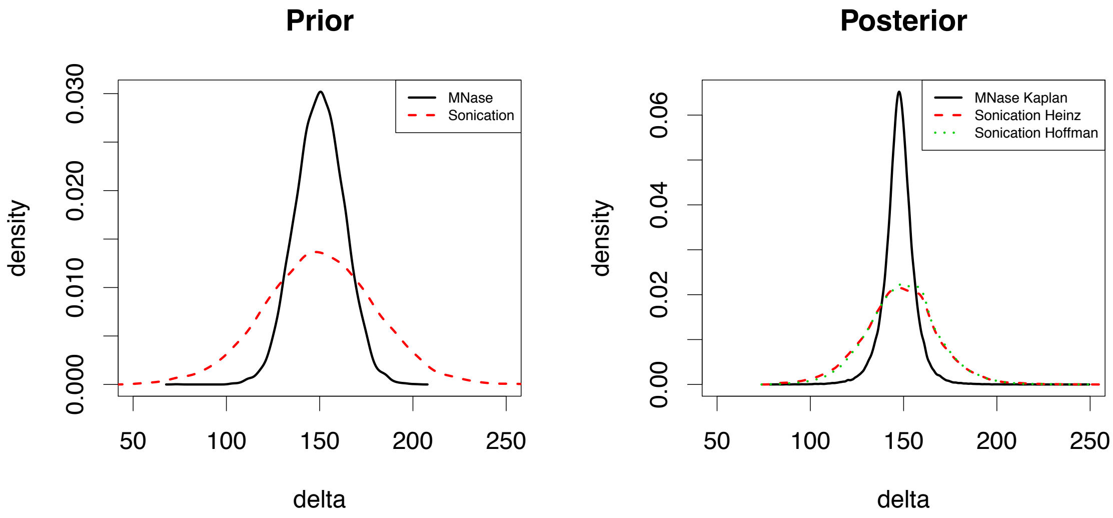

Figure 2. Left: Prior density of δ with selected values for hyperparameters for MNase-based and sonicated ChIP-seq data. Right: Posterior density of δ in Kaplan (2009), Heinz (2010) and Hoffman (2010) data sets.

2. Score curves of PING, NPS, and template filter

The figures shows the distributions of scores for PING, TemplateFilter (TF) and NPS nucleosome predictions for different data sets, with a vertical line showing an estimated ‘elbow’ point of the curves. Each method's scores have been rescaled to the range [0,1].

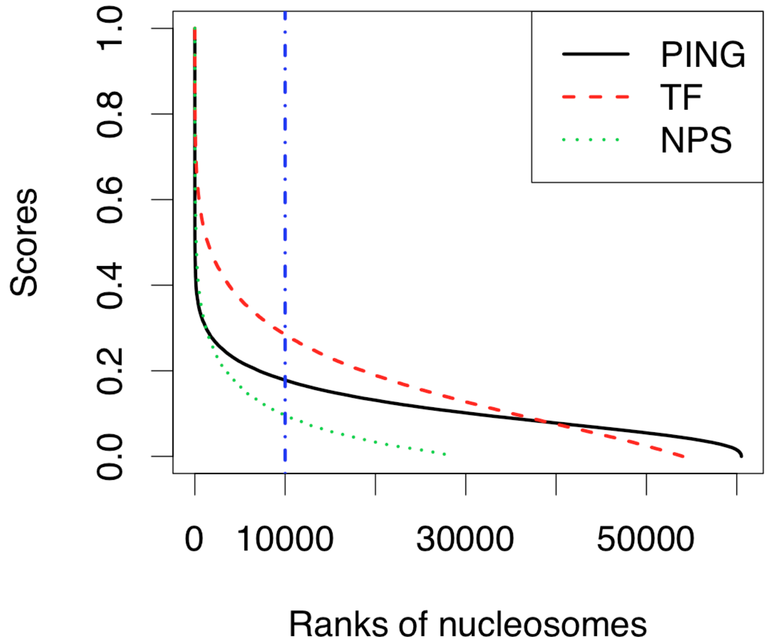

Figure 3. Nucleosome prediction scores for PING, TemplateFilter (TF) and NPS for MNase-seq NOCL_R4 data (Kaplan 2009). The vertical line marks the top-ranked 10,000 predicted nucleosomes for each method.

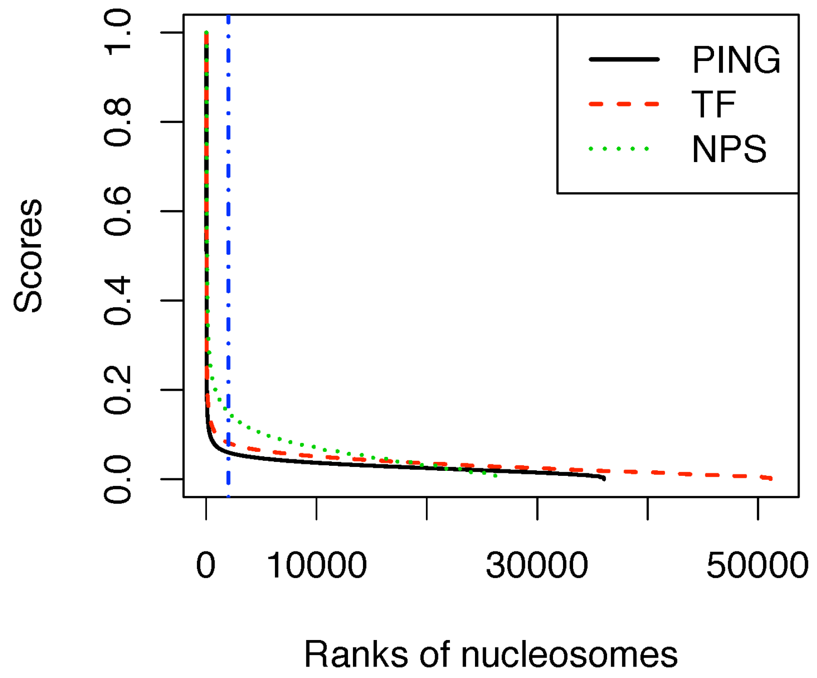

Figure 4. Nucleosome prediction scores for PING, TemplateFilter (TF) and NPS, for sonicated H3K4me1 ChIP-seq data from mouse PUER cells, 1 hour after stimulation (Heinz 2010). The vertical line marks the top-ranked 2000 predicted nucleosomes for each method.

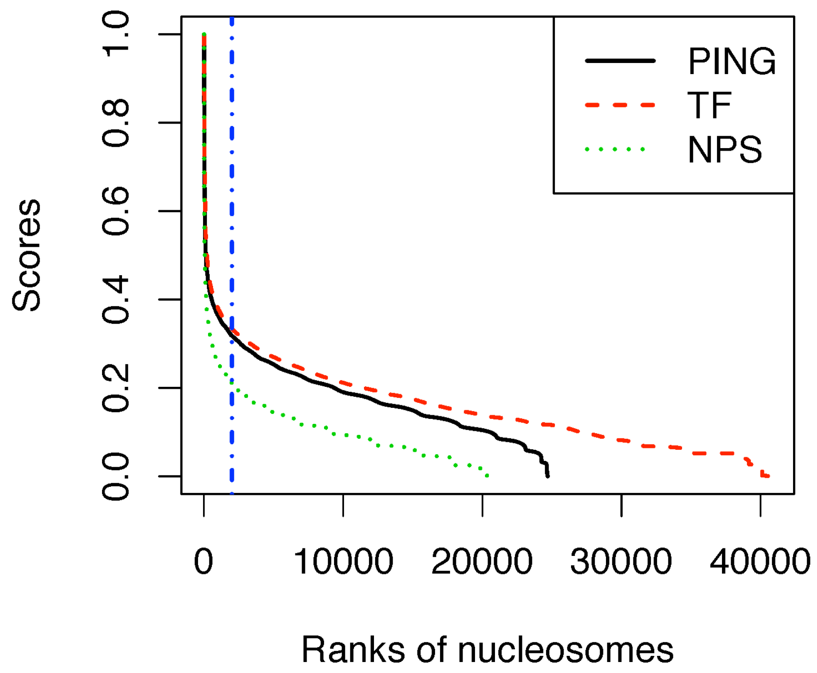

Figure 5. Nucleosome prediction scores for mouse islet sonicated H3K4me1 ChIP-seq data (Hoffman 2010). The vertical line marks the top-ranked 2000 predicted nucleosomes for each method.

3. Testing local enrichment differences between neighboring nucleosomes

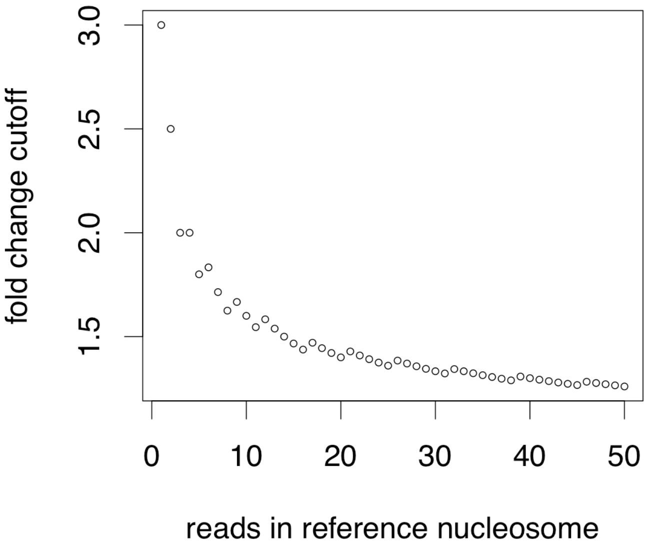

Figure 6. Adaptive threshold for local enrichment, based on the number of reads and the peak width of both nucleosomes, obtained from the negative binomial (NB) model. The parameter 'prob' in the NB model is assumed to be 0.5 in this figure. To calculate the adaptive threshold the significance level used is 0.1 (i.e. the 90% quantile in the NB distribution).

4. PING scores of all predicted nucleosomes in islet and liver H3K4me1 data

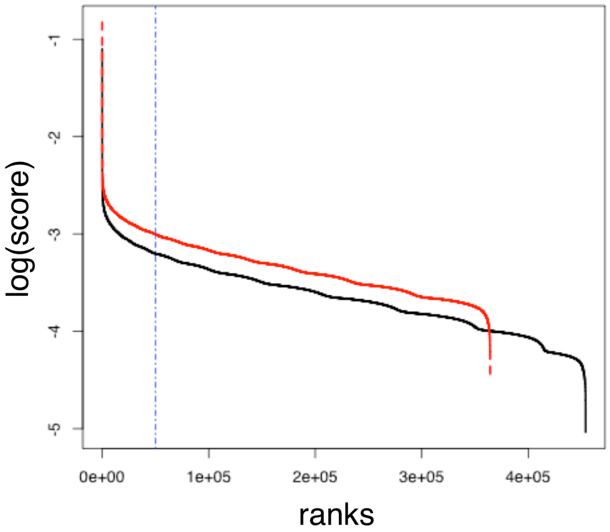

Figure 7. Sorted PING scores for all nucleosomes predicted in mouse islets (black) and liver (red) from sonicated ChIP-seq data (Hoffman 2010). The vertical dotted blue line shows the top-ranked 50000 nucleosomes from each data set.

5. Modality and nucleosome occupancy for Foxa2 and Pdx1 binding sites in mouse adult liver tissue

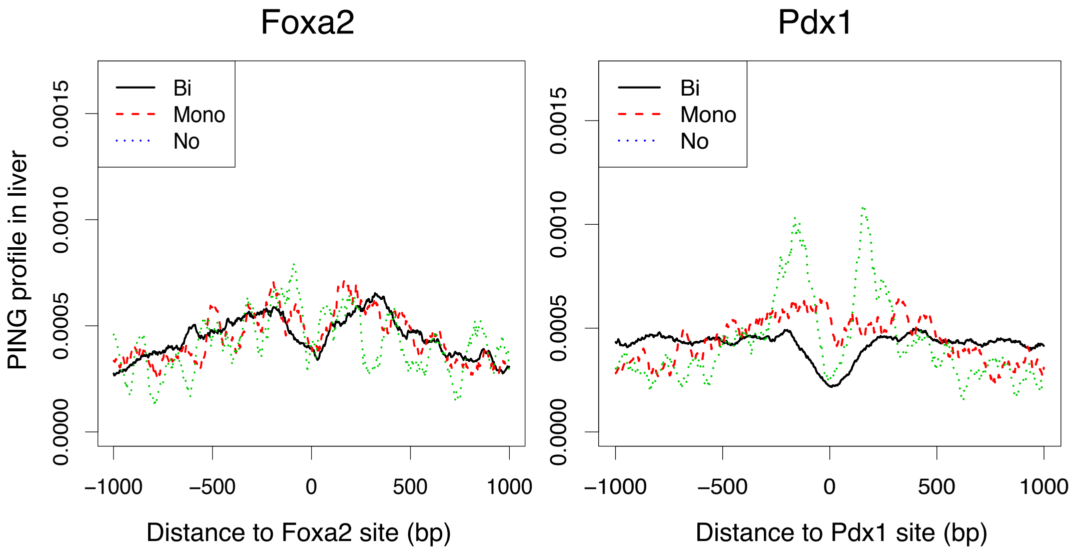

Figure 8. Average model-based nucleosome positioning profiles for Foxa2 and Pdx1 binding sites identified using ChIP-seq data. Compare to Fig. 3. Of main manuscript Profiles show results for mouse adult liver tissue from (Hoffman 2010) for bimodal (solid black lines), monomodal (dashed red lines) and NoNuc (dotted green lines) binding sites. A NoNuc transcription factor binding site had no H3K4me1-marked nucleosome prediction within 1 kb of its peak summit, a monomodal site had at least one nucleosome prediction within 50 bp of its summit, and all other sites were bimodal.

6. Performance of a naive method on ChIP-seq data

Naive methods can generate well-defined nucleosome occupancy profiles for MNase-based data (e.g. Kaplan 2009 and Heinz 2010). Such methods generate a pileup depth profile from reads that have been extended by, say, 146 bp. Comparing Figure 2B of main manuscript with the figure below, in which no nucleosome positions can be seen in the profiles, shows that PING is more effective for interpreting ChIP-seq data.

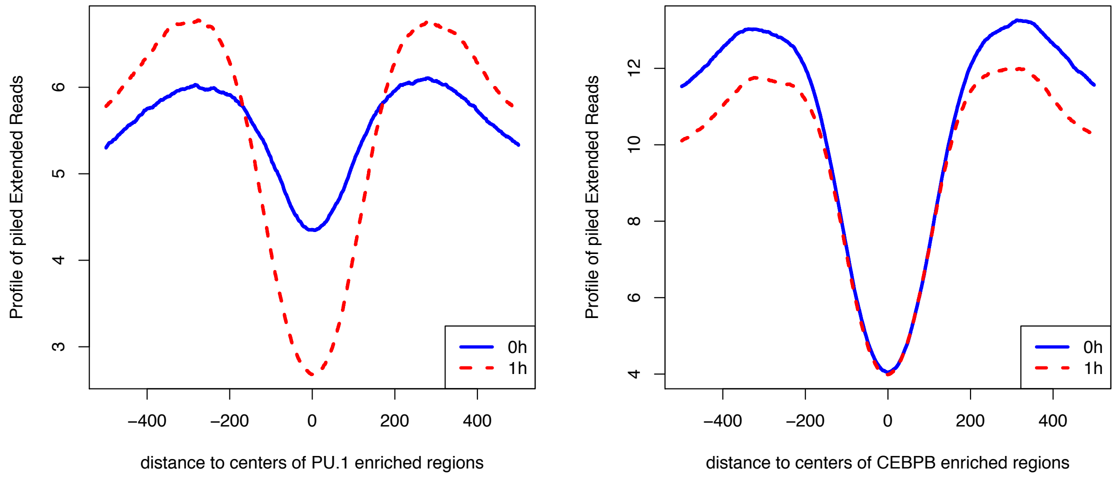

Figure 9. Noninformative nucleosome occupancy profiles generated from ChIP-seq H3K4me1 data using an extended-read pileup method. Compare to Fig. 2B of main manuscript, which shows PING profiles generated from the same data.
