# Supplementary material for: Probabilistic Inference for Nucleosome Positioning with MNase-Based or Sonicated Short-Read Data
Source: PLoS One. 2012 Feb 29;7(2):e32095. doi: 10.1371/journal.pone.0032095 (PMC3290535; doi:10.1371/journal.pone.0032095)
Supplement: Table S1 — Contains the supplementary table referred to in the main manuscript. (PDF) [file pone.0032095.s002.pdf]

Number of predicted nucleosomes from PING, NPS, and TemplateFilter

Supplementary Table 1 The number (in thousands) of predicted nucleosomes for NOCL4 data (Kaplan 2009), 5000 selected regions of (Heinz 2010)'s 1hr H3K4me1 data, 5000 selected regions (Hoffman 2010)'s islet and their random subsets of reads using PING, TemplateFilter and NPS.

Data	% subsets	100	95	90	85	80	75	70	65	60	55	50	45	40	35	30
Kaplan NOCL4	PING	61	60	60	60	59	59	58	58	57	57	56	55	54	53	51
	TemplateFilter	54	54	54	54	54	54	54	54	55	55	54	54	55	55	55
	NPS	28	28	27	27	26	26	25	24	23	22	21	21	20	18	15
Heinz's 1 hour	PING	36	35	34	33	32	31	30	28	26	24	22	20	17	14	11
	TemplateFilter	51	50	50	49	48	47	46	44	43	42	40	38	36	33	30
	NPS	27	26	26	25	24	24	23	22	21	20	19	18	17	15	13
Hoffman's islets	PING	25	24	23	21	20	19	17	16	14	12	10	8	6	4	2
	TemplateFilter	41	40	39	38	37	36	35	33	32	30	28	26	24	22	19
	NPS	20	20	19	18	18	17	16	15	14	13	12	11	9	7	6
