# Supplementary material for: Probabilistic Inference for Nucleosome Positioning with MNase-Based or Sonicated Short-Read Data
Source: PLoS One. 2012 Feb 29;7(2):e32095. doi: 10.1371/journal.pone.0032095 (PMC3290535; doi:10.1371/journal.pone.0032095)
Supplement: Text S1 — Describes details of methods, and discusses PING's parameters. (PDF) [file pone.0032095.s003.pdf]

Supplementary text for Probabilistic Inference for Nucleosome Positioning with MNase-based or Sonicated Short-read Data

January 26, 2012

1 Derivation of the EM algorithm

Introduce latent variables

To use the EM algorithm to fit the model, we introduce the latent variables $\boldsymbol{z}$'s and $\boldsymbol{u}$'s, and rewrite our hierarchical t-mixture models as

$$\begin{aligned}
\boldsymbol{z}_{fi}, \boldsymbol{z}_{rj} &\sim \text{Multinomial(w)}, & (1)\\
(f_i | U_{fik} = u_{fik}, z_{fik} = 1, \mu_k, \delta_k) &\sim \text{N}\left(\mu_k + \delta_k/2, \sigma^2_{fk}/u_{fik}\right) & (2)\\
(r_j | U_{rjk} = u_{rjk}, z_{rjk} = 1, \mu_k, \delta_k) &\sim \text{N}\left(\mu_k - \delta_k/2, \sigma^2_{rk}/u_{rjk}\right) & (3)\\
(U_{fik} | z_{fik} = 1), (U_{rjk} | z_{rjk} = 1) &\sim \mathcal{G}a(v/2, v/2). & (4)
\end{aligned}$$

The unknown membership indicator $\boldsymbol{z}_{fi}$ (resp. $\boldsymbol{z}_{rj}$) indicates which nucleosome a forward read i (resp. reverse read j) is associated with; both $\boldsymbol{z}_f$ and $\boldsymbol{z}_r$ share the same multinomial distribution given by (1). Conditional on the memberships (the $\boldsymbol{z}$'s), the read positions follow a t-distribution. We introduce another latent variable $\boldsymbol{u}$, and rewrite the t-distribution as a Normal-Gamma compound distribution given by (2 - 4).

Conditional expectation of the penalized log complete data likelihood

From this point, for ease of notation, we use the letter d to denote either f or r. Let us denote the unknown parameter vector by $\boldsymbol{\Theta} = (\boldsymbol{\theta}_1, \ldots, \boldsymbol{\theta}_K)$ where $\boldsymbol{\theta}_k = (w_k, \mu_k, \delta_k, \sigma^2_{fk}, \sigma^2_{rk})$, and the complete data by $\boldsymbol{c} = (\boldsymbol{y}_f, \boldsymbol{y}_r, \boldsymbol{z}_f, \boldsymbol{z}_r, \boldsymbol{u}_f, \boldsymbol{u}_r)$. Then the complete data log-likelihood can be written as

$$\begin{aligned}
l(\boldsymbol{\Theta}|\boldsymbol{c}) &= \sum_{i=1}^{n_f}\sum_{k=1}^{K} z_{fik}\left\{\log\left[w_k \text{N}\left(y_{fi} \,\middle|\, \mu_k - \frac{\delta_k}{2}, \frac{\sigma^2_{fk}}{u_{fik}}\right)\right] + \frac{\nu}{2}(\log u_{fik} - u_{fik}) - \log u_{fik}\right\}\\
&+ \sum_{j=1}^{n_r}\sum_{k=1}^{K} z_{rjk}\left\{\log\left[w_k \text{N}\left(y_{rj} \,\middle|\, \mu_k + \frac{\delta_k}{2}, \frac{\sigma^2_{rk}}{u_{rjk}}\right)\right] + \frac{\nu}{2}(\log u_{rjk} - u_{rjk}) - \log u_{rjk}\right\}\\
&+ (n_f + n_r)\left[\frac{\nu}{2}\log\left(\frac{\nu}{2}\right) - \log\Gamma\left(\frac{\nu}{2}\right)\right].
\end{aligned}$$

Similarly, we define the prior penalty l_{prior} as

$$\begin{aligned} l_{\text{prior}} &= \sum_{k=1}^{K} \log \pi(\delta_k, \sigma_{rk}^2, \sigma_{fk}^2, \mu_k | \rho, \xi, \alpha, \beta, \lambda) \\ &= \sum_{k=1}^{K} \left\{ \frac{1}{2} \log(\sigma_{rk}^{-2} + \sigma_{fk}^{-2}) - \frac{\rho}{2}(\sigma_{fk}^{-2} + \sigma_{rk}^{-2})(\delta - \xi)^2 \right\} - \lambda \sum_{k=1}^{K-1} (\mu_{k+1} - \mu_k - 200)^2 \\ &\quad + \sum_{k=1}^{K} \left\{ (\alpha - 1) \log(\sigma_{fk}^{-2}) - \beta \sigma_{fk}^{-2} + (\alpha - 1) \log(\sigma_{rk}^{-2}) - \beta \sigma_{rk}^{-2} \right\} + \text{cst} \\ &= -\frac{1}{2} \sum_{k=1}^{K} \left\{ (\sigma_{fk}^{-2} + \sigma_{rk}^{-2})[\rho(\delta_k - \xi)^2 + 2\beta] - (2\alpha - 1) \log(\sigma_{fk}^{-2} + \sigma_{rk}^{-2}) \right\} \\ &\quad - \lambda \sum_{k=1}^{K-1} (\mu_{k+1} - \mu_k - 200)^2 + \text{cst} \end{aligned}$$

where cst is a constant.

Given the current estimate $\mathbf{\Theta}^-$ for $\mathbf{\Theta}$, the conditional expectation of the penalized log complete data likelihood is given as

$$\begin{aligned} Q(\mathbf{\Theta}|\mathbf{\Theta}^-) &\stackrel{\text{def}}{=} \mathbb{E}[l(\mathbf{\Theta}|\boldsymbol{c})|\mathbf{\Theta}^-] + l_{\text{prior}} \\ &= \sum_{d \in \{f,r\}} \sum_{i=1}^{n_d} \sum_{k=1}^{K} \widetilde{z}_{dik} \left\{ \log w_k - \log \sigma_{dk} - \frac{\widetilde{u}_{dik}}{2} \left(\frac{d_i - \mu_{dk}}{\sigma_{dk}} \right)^2 \right\} + l_{\text{prior}} + \text{cst}. \quad (5) \end{aligned}$$

E-Step

The E-step (Peel and McLachlan, 2000) consists of computing the following quantities:

$$\widetilde{z}_{dik} \stackrel{\text{def}}{=} \mathbb{E}(Z_{dki}|\boldsymbol{y}_{di}, \mathbf{\Theta}^-) = \frac{w_k t_4(d_i|\mu_{dk}, \sigma_{dk})}{\sum_{k=1}^{K} w_k t_4(d_i|\mu_{dk}, \sigma_{dk})}, \quad (6)$$

$$\widetilde{u}_{dik} \stackrel{\text{def}}{=} \mathbb{E}(U_{dik}|\boldsymbol{y}_{di}, z_{dik} = 1, \mathbf{\Theta}^-) = \frac{\nu + 1}{\nu + (d_i - \mu_{dk})^2/\sigma_{dk}^2}. \quad (7)$$

M-Step

During the M-step, the goal is to maximize $Q(\mathbf{\Theta}|\mathbf{\Theta}^-)$ with respect to $\mathbf{\Theta}$, which requires solving $\partial Q(\mathbf{\Theta}|\mathbf{\Theta}^-)/\partial \mathbf{\Theta} = \mathbf{0}$. We solve the following equation system to obtain updated parameter

estimates

$$0 = \frac{\partial Q^*}{\partial \widehat{w}_k} = \sum_{d\in\{f,r\}} \sum_{i=1}^{n_d} \sum_{k=1}^{K} \widetilde{z}_{dik} w_k^{-1}, \quad \sum_{k=1}^{K} w_k = 1 \tag{8}$$

$$0 = \frac{\partial Q^*}{\partial \widehat{\mu}_k} = \sum_j \widetilde{z}_{rjk}\widetilde{u}_{rjk} \frac{(y_{rj} - \widehat{\mu}_k - \frac{\widehat{\delta}_k}{2})}{\widehat{\sigma}^2_{rk}} + \sum_i \widetilde{z}_{fik}\widetilde{u}_{fik} \frac{(y_{fi} - \widehat{\mu}_k + \frac{\widehat{\delta}_k}{2})}{\widehat{\sigma}^2_{fk}} + q_k \tag{9}$$

$$\begin{aligned} 0 = \frac{\partial Q^*}{\partial \widehat{\delta}_k} &= \sum_j \widetilde{z}_{rjk}\widetilde{u}_{rjk} \frac{(y_{rj} - \widehat{\mu}_k - \frac{\widehat{\delta}_k}{2})}{2\widehat{\sigma}^2_{rk}} - \sum_i \widetilde{z}_{fik}\widetilde{u}_{fik} \frac{(y_{fi} - \widehat{\mu}_k + \frac{\widehat{\delta}_k}{2})}{2\widehat{\sigma}^2_{fk}} \\ &\quad -\rho(\widehat{\sigma}^{-2}_{fk} + \widehat{\sigma}^{-2}_{rk})(\widehat{\delta}_k - \xi) \end{aligned} \tag{10}$$

$$0 = \frac{\partial Q^*}{\partial \widehat{\sigma}^{-2}_{fk}} = \sum_i \frac{\widetilde{z}_{fik}}{2} \left[\widehat{\sigma}^2_{fk} - (y_{fi} - \widehat{\mu}_k + \frac{\widehat{\delta}_k}{2})^2 \widetilde{u}_{fik} \right] - \frac{\rho(\widehat{\delta}_k - \xi)^2 + 2\beta}{2} + \frac{2\alpha - 1}{2}\widehat{\sigma}^2_{fk} \tag{11}$$

$$0 = \frac{\partial Q^*}{\partial \widehat{\sigma}^{-2}_{rk}} = \sum_j \frac{\widetilde{z}_{rjk}}{2} \left[\widehat{\sigma}^2_{rk} - (y_{rj} - \widehat{\mu}_k - \frac{\widehat{\delta}_k}{2})^2 \widetilde{u}_{rjk} \right] - \frac{\rho(\widehat{\delta}_k - \xi)^2 + 2\beta}{2} + \frac{2\alpha - 1}{2}\widehat{\sigma}^2_{rk}, \tag{12}$$

where q_k comes from the Gaussian Markov Random Field prior assigned to μ_k, and is given by

$$q_k = \begin{cases} -2\lambda(2\hat{\mu}_k - (\hat{\mu}_{k-1} + \hat{\mu}_{k+1})), & \text{if } k \neq 1, K, \\ -2\lambda(\hat{\mu}_1 - \hat{\mu}_2 + 200), & \text{if } k = 1, \\ -2\lambda(\hat{\mu}_K - \hat{\mu}_{K-1} - 200), & \text{if } k = K. \end{cases}$$

Because there is no simple closed form solution for $\boldsymbol{\Theta}$, we adopted a conditional approach in which we first maximize over $(\mathbf{w}, \boldsymbol{\mu}, \boldsymbol{\delta})$, conditional on $(\boldsymbol{\sigma}_f, \boldsymbol{\sigma}_r)$, and then maximize over $(\boldsymbol{\sigma}_f, \boldsymbol{\sigma}_r)$, conditional on the previously updated $(\mathbf{w}, \boldsymbol{\mu}, \boldsymbol{\delta})$, resulting in an Expectation/Conditional Maximization (ECM) algorithm (Meng and Rubin, 1993). Conditionally on σ_{fk} and σ_{rk}, the parameters $(\widehat{w}_k, \widehat{\mu}_k, \widehat{\delta}_k)$ are updated by the unique solution of the linear system given by (8 - 10). Similarly, conditionally on these new estimates $\widehat{w}_k, \widehat{\mu}_k, \widehat{\delta}_k$, we can then solve a linear system given by (11 and 12).

2 Post-processing PING results

Given the range of cases that short-read data present to PING's mixture modeling, we detect and post-process certain problematic events within the results that the mixture model returns.

2.1 Mismatched peaks

When one of a nucleosome's forward/reverse density profile peaks is much more asymmetric and/or taller than the other, mixture models may use different number of components to describe the F peak and R peaks of the same nucleosome. In such a case, unmatched mixture components within a nucleosome will be matched to components of other nucleosomes, which will cause subsequently processed nucleosomes to have mismatched components and incorrectly estimated center locations. We detect such problems as atypical estimated δ outside of the range of $(80, 250)$, or as un-fittable models, and fit a new model to such a candidate region, using a stronger prior on δ. For such a new prior we set $\rho = 8$, which reduces the prior's standard error

to be in the range $(7, 16)$. While this strong prior helps address the mismatch problem, it is too strong to be used globally, as the range of true δ is much wider than it permits.

2.2 Missing nucleosomes

A nucleosome may have much higher F/R density profile peaks than its neighbouring nucleosomes in the same candidate region. In such a case, mixture models tend to use many components to describe the nucleosome with tall peaks, while using a single component to describe multiple low-peak nucleosomes, and PING can fail to detect some low rank nucleosomes. We can detect such problems by looking for predictions with forward and reverse peaks that are too wide ($\sigma_f, \sigma_r > 50$ for MNase, and $\sigma_f, \sigma_r > 70$ for sonication), then extracting reads within a distance of 2σ from the estimated peak centers, and refitting the model with these reads. This is typically effective, since reads from higher peak are excluded in the new fitted model, and new hyper-parameters $\alpha = 100$ and $\beta = 100000$ are used to ensure that the 95% CI of prior σ is $(20, 25)$.

2.3 Duplicate predictions

When we divide a long candidate region into shorter regions, some care is needed. The concern arises because, when a nucleosome is near the cutting point, enough reads could be present in each of the new candidate regions that the same nucleosome can be predicted in both of the new candidate regions. To address this, when we find predicted nucleosomes that are in adjacent candidate regions and are within 100 bp of each other, we extract reads from and between these two nucleosomes and refit the model. We let the new fitted model decide whether or not to merge the duplicates into a single nucleosome prediction or to retain both predictions.

3 PING's parameters

In this section, we summarize PING's parameters, giving default/suggested values and describing the effects of changing these values.

3.1 Segmentation parameters

Overall, the tuning parameters used in the segmentation step have very little effect on the final results. This being said, for clarity, we describe each parameter and its effect on the model, as follows.

- $w = 300$. The default width of the sliding window is 300 bp. Small changes in the window width have minimal effects on the locations of predicted nucleosomes. We recommend users change the default value only when a large proportion of DNA fragments in their experiment are longer than 300 bp.
- $s = 2$. The default sliding window step size is 2 bp. While changing this value will change the resolution of segmentation step, hence might change the boundaries of candidate regions, we assessed values ranging from 1 to 5 and saw only minor changes. A value of $s = 1$ could be used at the cost of increasing the overall computing time.

- $n = 5$. A sliding window is retained if at least 5 forward and 5 reverse reads are respectively observed in the left and right halves of the sliding window. Using a smaller value of n should return more low-rank nucleosome predictions. These will require more computing time for model fitting, but will have lower enrichment and confidence levels, hence may be less useful. In any case, we recommend using a value of n larger than 2 to ensure that all regions have enough reads to estimate the model parameters. High-rank predicted nucleosomes (i.e. the nucleosomes of interest, true positives) will not be affected by slight changes in n.

3.2 Model fitting parameters

The hyperparameters of the prior distribution were chosen empirically based on prior knowledge about the data sets described in this work and other similar studies. We first chose the mean of the prior distribution for δ, namely ξ, which can be seen as our best estimate of the average fragment length, and then chose the other hyperparameters so that the variability around this prior guess matched our prior knowledge. This resulted in the following parameters for the two type of datasets used here:

- For MNase-seq data, we use $\alpha = 20$, $\beta = 20000$, $\rho = 3$, and $\xi = 150$, which results in estimated lengths of DNA fragments, δ, of between 100 and 200 bp.
- For ChIP-seq data, we use $\alpha = 10$, $\beta = 20000$, $\rho = 1.2$, and $\xi = 150$, which results in δ values between 50 and 250 bp.

These priors are mildly informative; slight changes in their values should have little effect on the resulting inference, especially for highly enriched nucleosomes.

References

McLachlan, G.J., and Jones, P.N. (1998) Fitting mixture models to grouped and truncated data via the em algorithm. *Biometrics* **44,** 571–578.

Meng, XL. and Rubin, D. (1993) Maximum likelihood estimation via the ECM algorithm: A general framework. *Biometrika* **80,** 267–278.

Peel, D. and McLachlan, G.J. (2000) Robust mixture modelling using the t distribution. *Statistics and Computing* **10,** 339–348.

Zhang, Y., Liu, T., Meyer, C.A., Eeckhoute, J., Johnson, D.S., Bernstein, B.E., Nussbaum, C., Myers, R.M., Brown, M., Li, W. and Liu, X.S. (2008) Model-based Analysis of ChIP-seq (MACS). *Genome Biology* **9,** R137.

Zhang X, Robertson G, Krzywinski M, Ning K, Droit A, Jones S, Gottardo R (2010) PICS: Probabilistic Inference for ChIP-seq. *Biometrics*, 2010.
