# Supplementary material for: Probabilistic Inference for Nucleosome Positioning with MNase-Based or Sonicated Short-Read Data
Source: PLoS One. 2012 Feb 29;7(2):e32095. doi: 10.1371/journal.pone.0032095 (PMC3290535; doi:10.1371/journal.pone.0032095)
Supplement: Examples S1 — A multi-page figure showing details of PING, NPS and TemplateFilter nucleosome calls in several genomic regions. (PDF) [file pone.0032095.s004.pdf]

## Examples of raw aligned reads and predicted nucleosomes

The figures in this file show examples of nucleosomes predicted using PING, NPS, and TemplateFilter in 2kb genomic regions from three different real data sets. Page 2 and 3 are generated from Hoffman's mouse sonicated ChIP-seq data. Page 4 and 5 are generated from Heinz's mouse sonicated ChIP-seq data. Page 6 and 7 are generated from Kaplan's yeast MNase-seq data.

Blue arrowheads represent aligned forward-strand reads; red show aligned reverse-strand reads. The curves shown above the reads are XSET profiles (blue for forward reads, red for reverse reads, black and gray considering all reads). The extension length for blue, red and black XSET profiles is the median of estimated $\delta$'s in the region, whereas the extension length of gray profile is fixed at 147 bp. The circles are predicted nucleosomes for (top to bottom) PING, NPS and TemplateFilter. The grayscale smears for PING predictions represent uncertainties for predicted nucleosome locations.

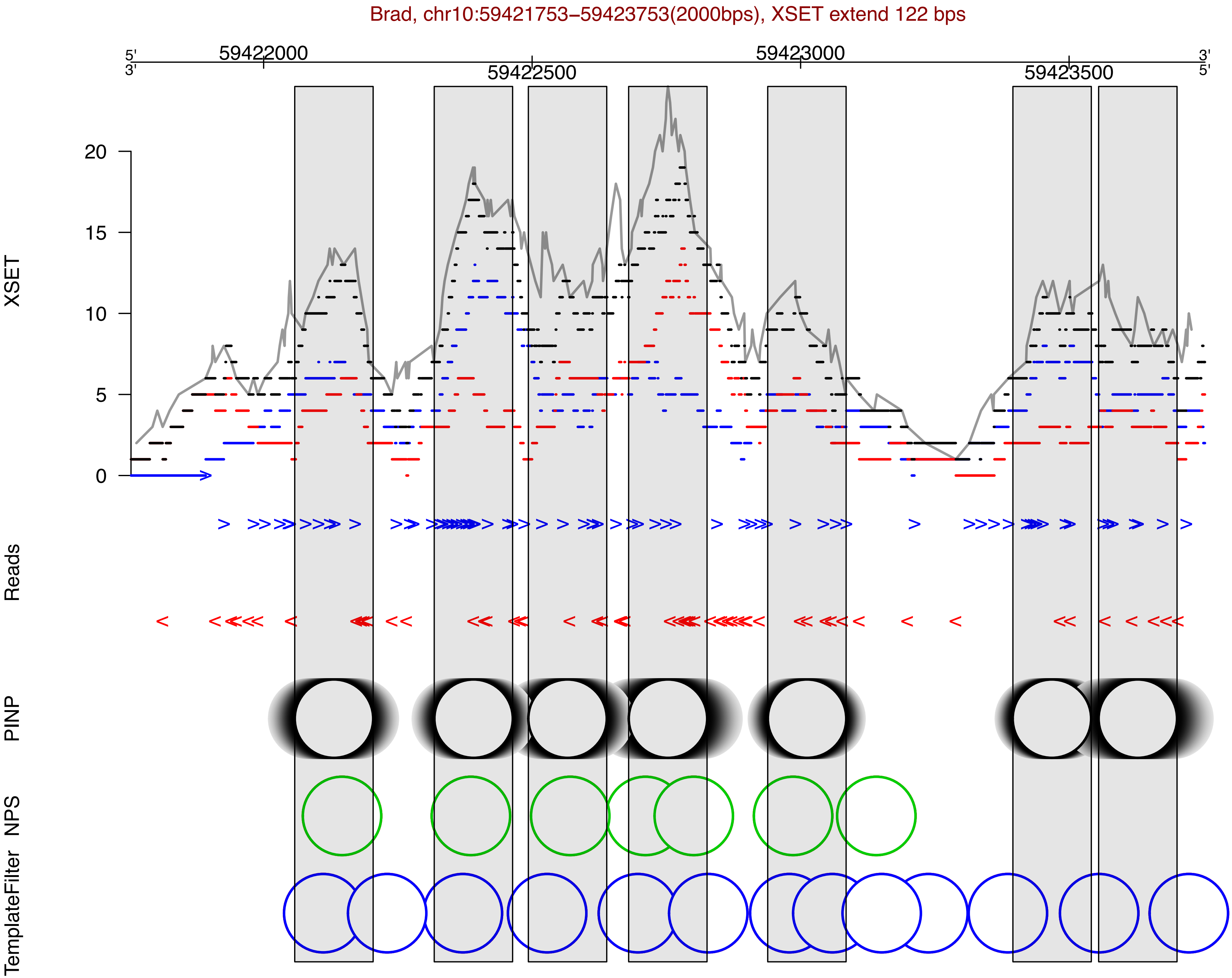

Brad, chr10:59421753–59423753(2000bps), XSET extend 122 bps
5'
3'
59422000
59422500
59423000
59423500
3'
5'
XSET
20
15
10
5
0
Reads
PINP
NPS
TemplateFilter

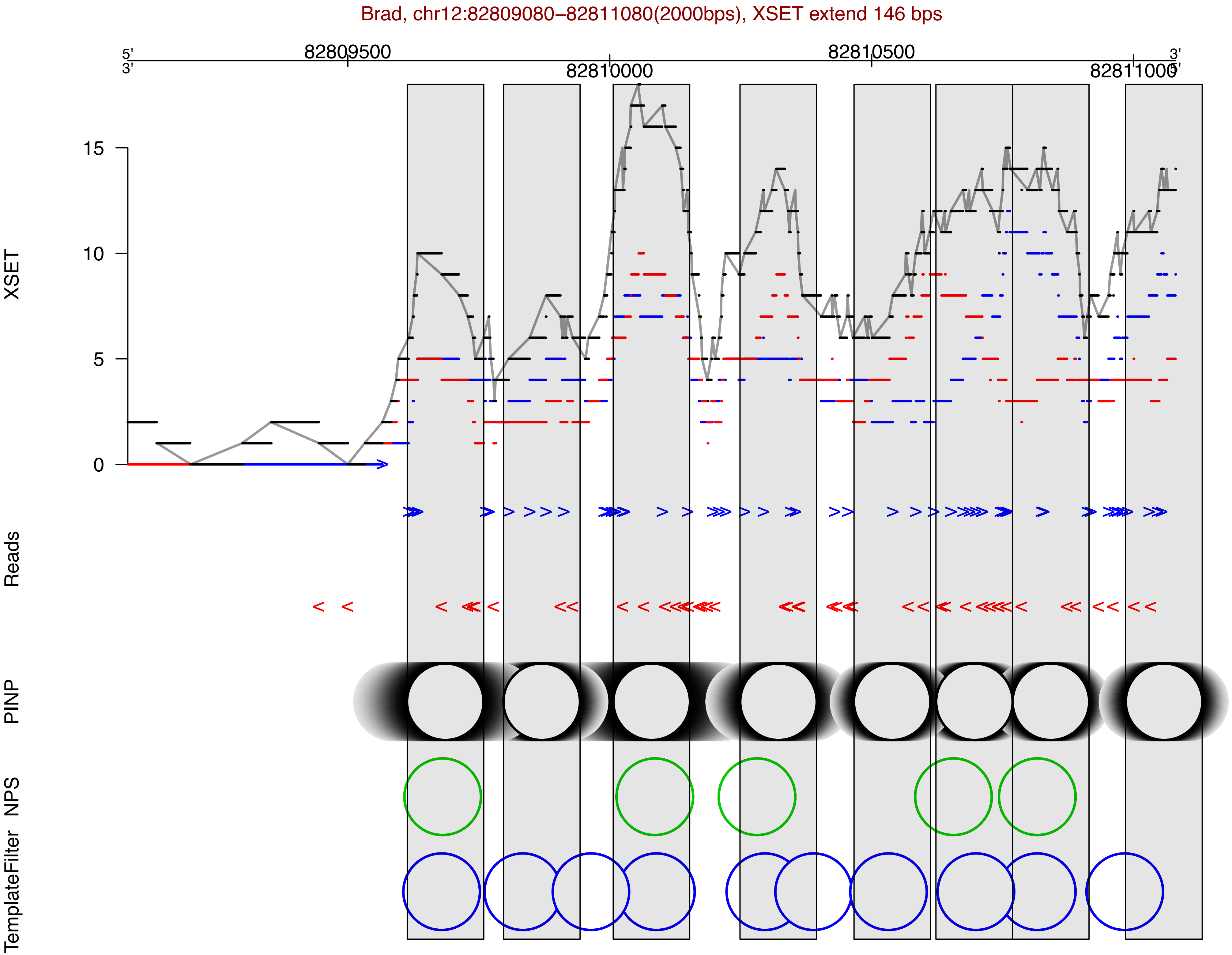

Brad, chr12:82809080–82811080(2000bps), XSET extend 146 bps
5'
3'
82809500
82810000
82810500
82811000
3'
5'
XSET
15
10
5
0
Reads
PINP
NPS
TemplateFilter

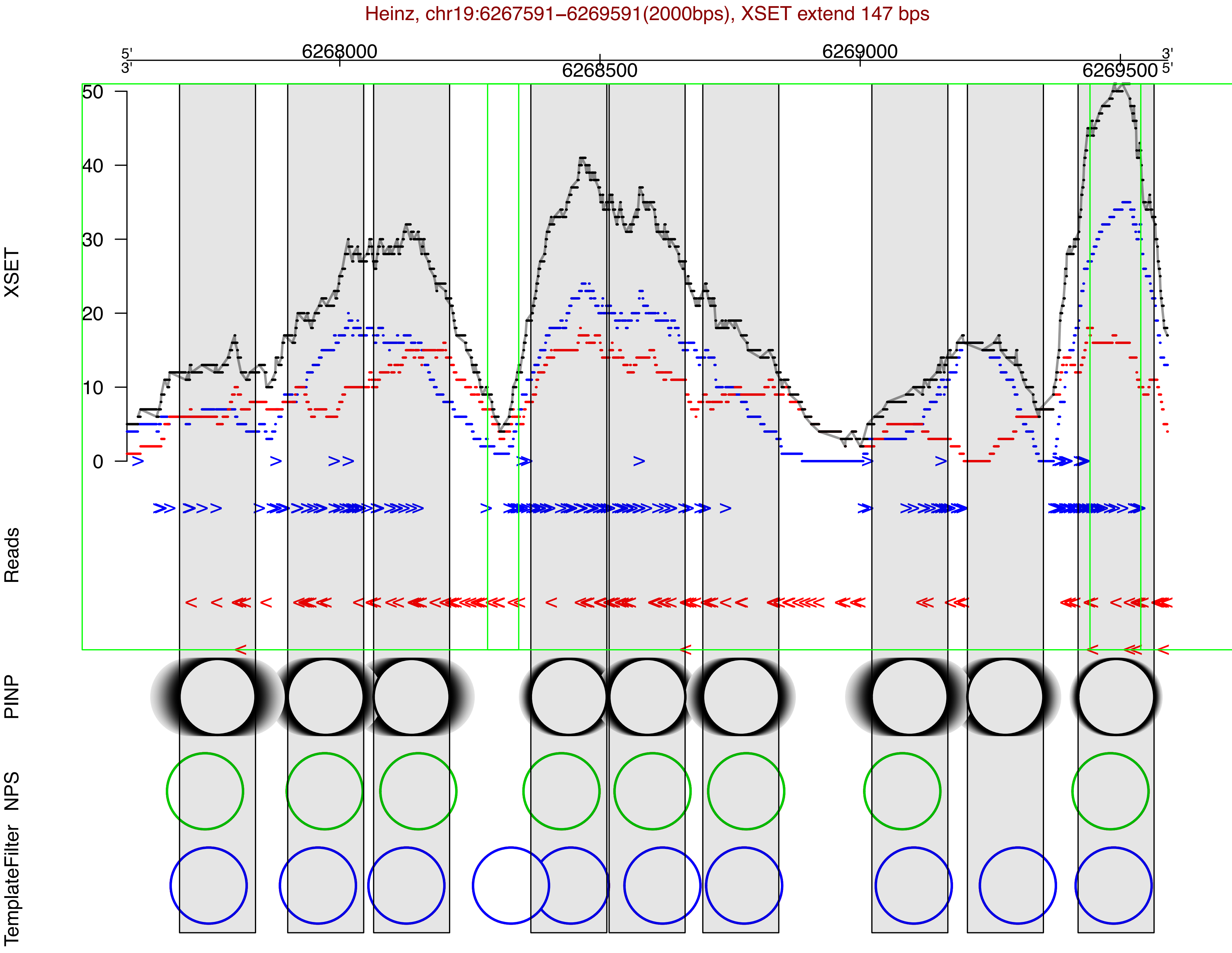

Heinz, chr19:6267591−6269591(2000bps), XSET extend 147 bps
5'
3'
6268000
6268500
6269000
6269500
3'
5'
50
40
30
20
10
0
XSET
Reads
PINP
NPS
TemplateFilter

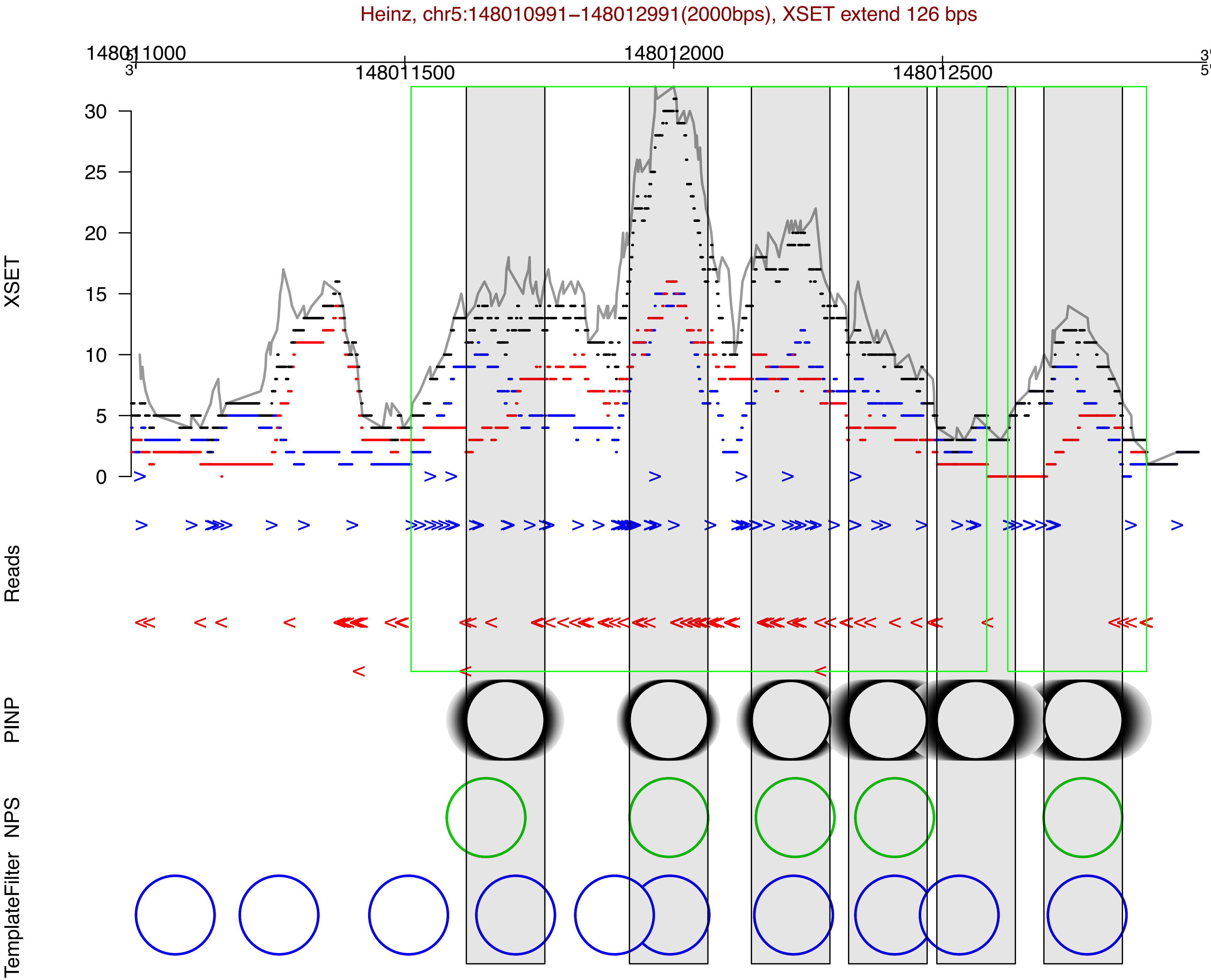

Heinz, chr5:148010991–148012991(2000bps), XSET extend 126 bps
148011000
148011500
148012000
148012500
5'
3'
XSET
30
25
20
15
10
5
0
Reads
PINP
NPS
TemplateFilter

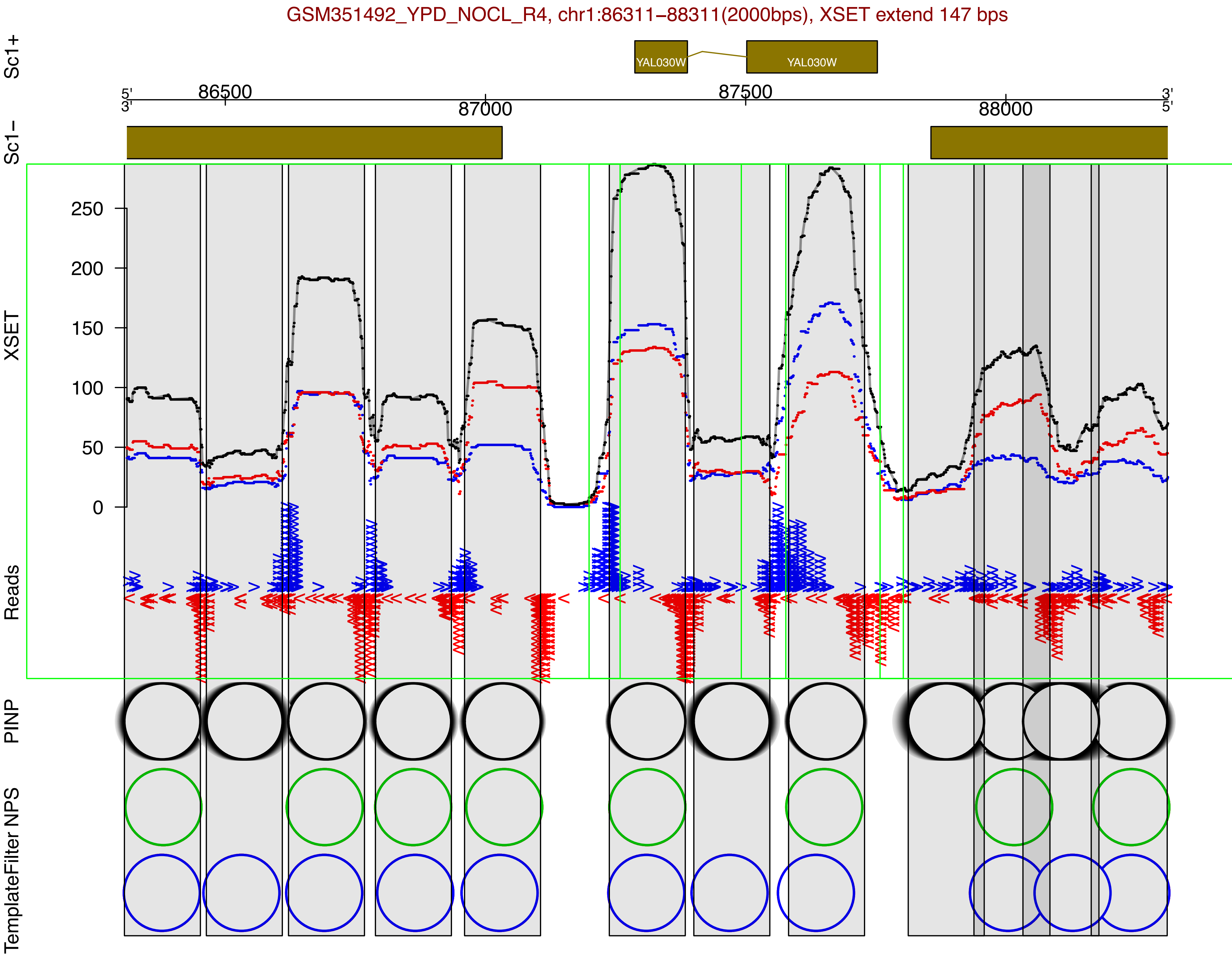

GSM351492_YPD_NOCL_R4, chr1:86311–88311(2000bps), XSET extend 147 bps
Sc1+
YAL030W
YAL030W
86500
87000
87500
88000
5'
3'
3'
5'
Sc1–
XSET
250
200
150
100
50
0
Reads
PINP
TemplateFilter NPS

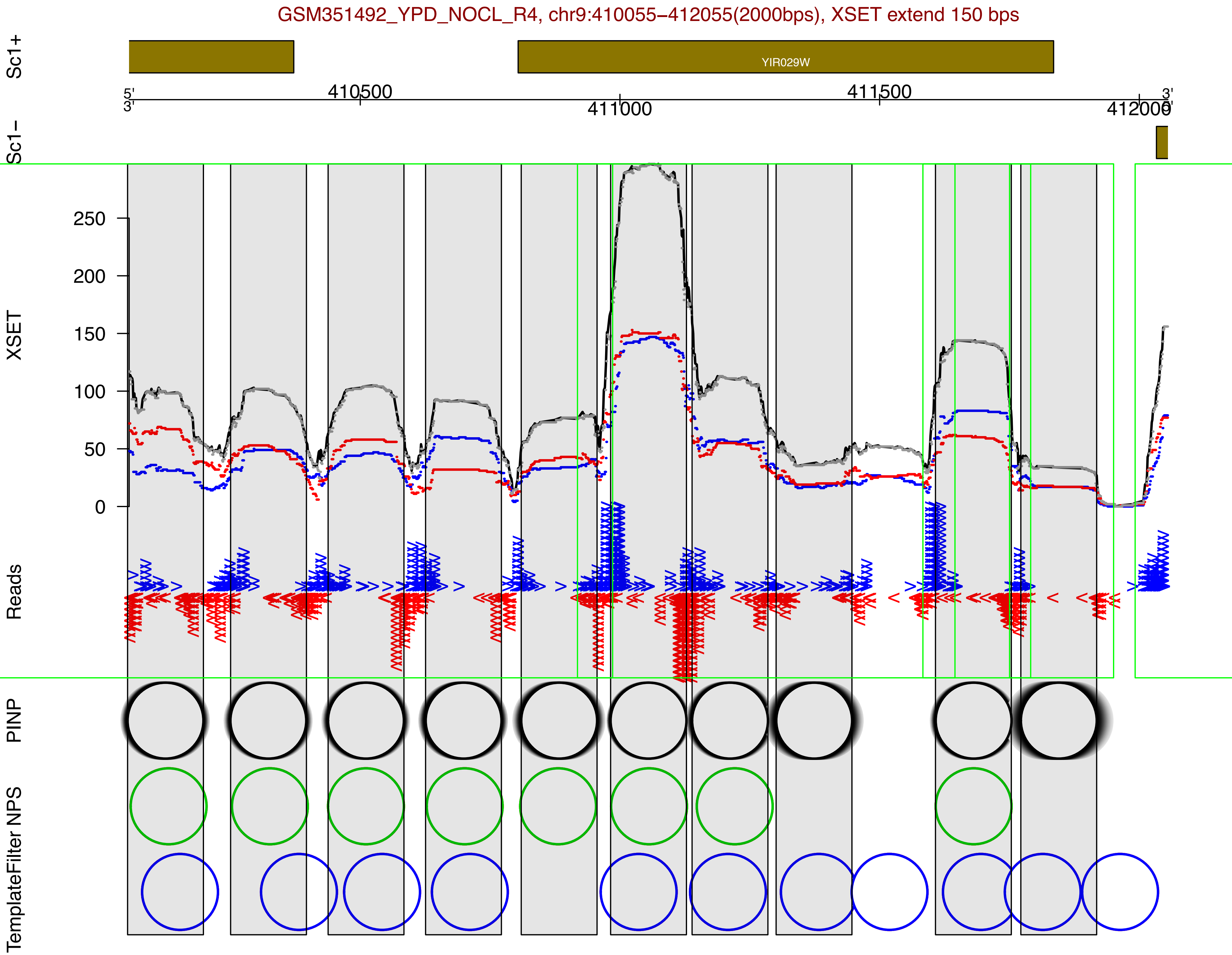

GSM351492_YPD_NOCL_R4, chr9:410055−412055(2000bps), XSET extend 150 bps
Sc1+
Sc1−
YIR029W
410500
411000
411500
412000
XSET
250
200
150
100
50
0
Reads
PINP
TemplateFilter NPS
